# Invariant Boltzmann-Shannon Entropy for Black-Holes: A Manifestly-Covariant Canonical Quantum-Gravity Approach

**DOI:** 10.3390/e28080932

**Published:** 2026-08-20

**Authors:** Claudio Cremaschini, Ramesh Radhakrishnan, Gerald Cleaver

**Affiliations:** 1 Research Center for Theoretical Physics and Astrophysics, Institute of Physics, Silesian University in Opava, Bezručovo nám.13, CZ-74601 Opava, Czech Republic; 2 Early Universe, Cosmology, and Strings (EUCOS) Group, Center for Astrophysics, Space Physics, and Engineering Research (CASPER), Waco, TX 76798, USA; ramesh_radhakrishna1@baylor.edu (R.R.); gerald_cleaver@baylor.edu (G.C.); 3 Department of Physics, Baylor University, Waco, TX 76798, USA

**Keywords:** covariant quantum gravity, manifest covariance, black-hole entropy, Boltzmann-Shannon entropy, quantum gravity PDF, Bekenstein-Hawking law

## Abstract

A novel theoretical study of Boltzmann-Shannon entropy arising in information-statistic theory applied to black-hole physics is proposed. The invariant setting implemented is represented by the manifestly-covariant quantum-gravity theory expressed in canonical Hamiltonian form. In such a framework the appropriate statistical interpretation relies on the configuration-space quantum expectation value of physical observables over the 4− scalar quantum-gravity probability density function (PDF). A representation for the black-hole Boltzmann-Shannon entropy is obtained for a Gaussian PDF profile and by establishing simultaneously a relationship between the black-hole invariant energy-content and the mean value of the quantum-gravity nonlinear Bohm potential. This yields a non-trivial functional dependence of the Boltzmann-Shannon entropy on the black-hole surface area, to be interpreted as a quantum statistical entropy counting black-hole bulk quantum-gravity states. The mathematical setting is shown to preserve manifest covariance and be self-contained within quantum-gravity realm. Comparisons with literature treatments dealing with thermodynamic or kinetic-statistical entropies that lead to the Bekenstein-Hawking black-hole surface entropy linear relation or its proposed quantum modifications are discussed.

## 1. Introduction

The peculiar nature of black-holes as mathematical solutions of space-time structure as well as possible real objects arising in high-energy astrophysical scenarios has been the subject of theoretical studies and speculative physical and logical investigations for a long time since their original proposal. Historically, the framework for the study of black-holes in classical physics is represented by General Relativity (GR), mathematical physics and field theory. Nevertheless, more recently a deeper interest in black-hole physics has grown in connection with quantum gravity. In a semi-classical description, this means regarding black-holes either as background sources of quantum gravitational fields or quantum-gravity phenomena affecting classical space-time. However, one can also envisage black-holes as purely-quantum exotic objects whose structure and dynamical behavior need to be disclosed by the appropriate quantum gravity theory, possibly subject to the constraint of generating the continuum classical framework as an emerging ensemble-average solution from microscopic quantum degrees of freedom. In this sense, black-holes could be unique scenarios for understanding the connection between classical and quantum gravitational field theories and test the correctness of GR itself and candidate quantum gravity settings [1,2,3,4,5].

In this reference, a breakthrough result that laid down the basis for the quantum treatment of black-holes was the discovery of their thermodynamic behavior, according to the semiclassical derivations originally proposed by Bekenstein [6,7] and Hawking [8,9,10]. This refers to the proof that black-holes can be regarded as black bodies with thermal radiation, and therefore endowed with the concepts of temperature and entropic laws. For a stationary axisymmetric Schwarzschild space-time these are expressed respectively by the Hawking temperature $T_{BH}$ and the Bekenstein-Hawking entropy $S_{BH}$. In particular, it was found that the latter entropy does not relate to the black-hole volume but rather to its surface, according to the area law relation expressed by

(1) $$S_{BH}=k_{B}\frac{A}{4\ell_{P}^{2}},$$

where $k_{B}$ is the Boltzmann constant, $A\equiv 4\pi R_{S}^{2}$ is the surface area of the black-hole with Schwarzschild radius $R_{s}\equiv \frac{2GM}{c^{2}}$ and invariant mass *M*, while $\ell_{P}\equiv \sqrt{\frac{G\hbar }{c^{3}}}$ is the Planck length, with *G*, *c* and *ℏ* being respectively the Newton gravitational constant, the speed of light in vacuum and the reduced Planck constant.

Historically, the result (1) was determined either by a thermodynamic approach or through statistical kinetic theory with additional constraint conditions on the state of particles and interaction with gravity [11]. In the latter case, an ensemble of test quantum particles surrounding the event horizon of a classical black-hole and involved in tunneling effect yielding so-called black-hole evaporation phenomena are usually considered, possibly under the assumption of validity of holographic principle. In the microscopic kinetic approach, one introduces the so-called partition function $W_{k_{B}}$ yielding the phase-space probability distribution that counts the accessible microstates of the system depending on its energy content *E*. The collective massive particles are assumed to be realized by non-relativistic bosons following Bose-Einstein statistics and obeying the ideal-gas energy-momentum relation. The logarithm of the partition function in such a case is given by

(2) $$\ln W_{k_{B}}=-\ln\left(1-e^{-\beta E}\right),$$

where $\beta \equiv \frac{1}{T}$, with *T* being the system statistical temperature. Then, in terms of $W_{k_{B}}$ and *E* the corresponding statistical kinetic entropy $S_{k}$ is determined as

(3) $$S_{k}=k_{B}\left(\ln W_{k_{B}}+\beta E\right),$$

where β represents a free parameter to be suitably determined. This indeterminacy is resolved by invoking statistical thermodynamic relations expressing the system pressure and energy density again in terms of $\ln W_{k_{B}}$ and then imposing that the latter coincides with the energy content of the black-hole, to be regarded as composed by the same ensemble of particles. This procedure permits linking the statistical solution, in particular the parameter β, to the characteristic properties of the black-hole, like its mass and Schwarzschild radius, when holographic principle is invoked [11].

Remarkably, also in the context of black-hole entropy, it was proved that the thermodynamic and statistical kinetic frameworks are equivalent, namely, they lead to the same result (1) for the entropy under validity of appropriate physical assumptions. For the analytical treatment of kinetic statistics, these include, for example, symmetry conditions inherited by the background metric solution and application of holographic principle [11], while relativistic kinetic and kinematic effects due to curved space-time as well as particle mutual interactions beyond ideal gas assumption are ignored within such approximation schemes. Finally, aspects related to the property of invariance of some concepts used in these derivations, like the energy one, and the covariance of the solutions with respect to the choice of particular metric tensor representations and reference frames remain questionable.

The Bekenstein-Hawking area law expressed by black-hole thermodynamical entropy is recognized as a cornerstone of classical GR and semiclassical treatments of quantum gravity effects trying to infer phenomenological aspects of quantum gravitational fields occurring in high-energy scenarios on the background of classical space-time solutions [12,13]. For this reason, the same entropy law became a reference test result for subsequent approaches attempting a comprehensive formulation of a plausible theory of quantum gravity and quantum establishment of black-hole entropy by disparate field theories. In this reference, an example is provided by the so-called fractal approach to describe the horizon geometry of black-holes postulated originally by Barrow and leading to the corresponding proposal of Barrow entropy $S_{d_{H}^{s}}$ generalizing the Bekenstein-Hawking solution [14]. The Barrow entropy is found to scale as $S_{d_{H}}∼A^{d_{H}^{s}/2}$, where $d_{H}^{s}$ is the so-called Hausdorff dimension, namely an appropriate parameter suitably related to the fractal black-hole. This generally yields a different power-law dependence of the entropy $S_{d_{H}^{s}}$ on the black-hole surface area with respect to the linear dependence expressed by (1).

Other attempts to the problem were elaborated instead within possibly full quantum regimes [15,16,17,18]. In the majority of cases the target carries a mathematical generalization of the area-law expression (1) with the inclusion of quantum corrections to be treated perturbatively. Among the most recognized settings in this direction, we recall string theory [19] and loop quantum gravity [20], as well as more recent approaches known as the holographic principle [21], the related AdS/CFT correspondence [22,23], the quantum relative entropy and the entanglement entropy arising for scalar fields near black-hole horizons [24,25,26,27], including the Ryu–Takayanagi conjecture [28]. It is worth mentioning that, in the framework of quantum description, a peculiar aspect of the solution that is common to disparate theories like string theory, supergravity, loop quantum gravity, noncommutative geometry and effective field theory is the prediction of a logarithmic correction of the area-law entropy in terms of the same black-hole surface area *A* [29,30,31,32]. This contribution arises as a sort of “fingerprint” of the underlying quantum treatment of black-hole entropy and appears as a transversal element yielding the scaling law S∼A+lnA. However, despite the huge number of investigations and proposals, the comprehension of entropic aspects of black-holes still represents a challenging target of theoretical physics.

In this paper, a novel approach to the problem is proposed, which is rooted on the theory of manifestly-covariant quantum gravity theory (CQG-theory) recently established [33,34]. This provides a framework for the comprehension of quantum gravity that is consistent with both mathematical and logical principles of classical and quantum physics. In particular, the following features of CQG-theory must be detailed in view of their relevance for the current research study:

(1) CQG-theory implements a formalism that is consistent with the Principle of Manifest Covariance, whereby all classical and quantum dynamical variables and observables are represented exclusively in 4−tensor form [35,36]. It is assumed that the space-time is represented by a Lorentzian differential manifold of the type $\left\{Q^{4},\hat{g}\left(r\right)\right\}$, with $Q^{4}$ being the 4−dimensional real vector space $R^{4}$ representing the space-time and $\hat{g}\left(r\right)\equiv \left\{{\hat{g}}_{\mu \nu }\left(r\right)\right\}\equiv \left\{{\hat{g}}^{\mu \nu }\left(r\right)\right\}$ being a real and symmetric metric tensor, which is parametrized with respect to a coordinate system (or GR-frame) $r\equiv \left\{r^{\mu }\right\}\in Q^{4}$.
(2) CQG-theory warrants the consistency with the deDonder-Weyl manifestly-covariant canonical representation formalism applying to continuum fields variational dynamics [37,38,39,40,41,42,43,44,45,46]. This permits to disclose the existence of a classical unconstrained Hamiltonian structure for the Einstein field equations. The latter is represented by the set $\left\{x_{R},H_{R}\right\}$, formed by the canonical state $x_{R}\left(s\right)\equiv \left(g\left(s\right),\pi \left(s\right)\right)$, with $g\left(s\right)\equiv \left\{g_{\mu \nu }\right\}\equiv \left\{g_{\nu \mu }\right\}$ and $\pi \left(s\right)\equiv \left\{\pi_{\mu \nu }\right\}\equiv \left\{\pi_{\nu \mu }\right\}$ being symmetric 4−tensor coordinate fields and momenta parametrized by an appropriate invariant proper-time *s*, and a suitable classical 4−scalar Hamiltonian density $H_{R}$. The corresponding Hamilton variational principle generates Hamilton equations that can be cast in evolution form in terms of the same evolution parameter *s*. These canonical equations are then proved to recover the Einstein field equations under prescription of suitable initial conditions for the initial canonical fields of the type
  (4) $$x_{R}\left(s_{o}\right)=\left(g_{\mu \nu }\left(s_{o}\right),\pi^{\mu \nu }\left(s_{o}\right)\right).$$
(3) CQG-theory determines a canonical quantization method of the classical Hamiltonian structure yielding a CQG-state identified with a single 4−scalar and complex function ψ(s) (CQG wave-function) of the form
  (5) $$\psi \left(s\right)\equiv \psi (g,\hat{g}\left(r\right),r\left(s\right),s),$$
  which spans a corresponding finite-dimensional Hilbert space endowed with scalar product. Upon introducing a Madelung representation for ψ(s), this means that the real function $\rho \left(s\right)\equiv \rho (g,\hat{g}\left(r\right),r\left(s\right),s)$ prescribed as
  (6) $$\rho \left(s\right)\equiv {\left|\psi (s)\right|}^{2}$$
  identifies the quantum probability density function (CQG-PDF) of $g\equiv \left\{g_{\mu \nu }\right\}$ on the configuration space $U_{g}$ with canonical measure $d\left(g\right)\equiv \prod_{\mu ,\nu =1,4}$$dg_{\mu \nu }$. Promoting the Poisson brackets to a quantum commutator then generates a 4−scalar CQG-quantum wave equation expressed by a Schroedinger-like first-order partial differential equation advancing in proper-time the 4−scalar quantum wave function ψ(s) and cast in conservative form, namely such that it warrants conservation of quantum probability.

These premises and the validity of CQG-theory provide the background for the present research on black-hole quantum entropy. More precisely, the theoretical framework is identified here with the Boltzmann-Shannon statistical entropy $S_{BS}$ (also known as Shannon entropy) that applies in information-statistic theories. The importance to implement notions of information theory to questions of entropic properties of black-hole physics and related logical paradoxes is gaining attention by the scientific community and has been recently recognized [23]. For example, the same setting was used in Ref. [47] to investigate the role of quantum entropy and establishment of H-theorems in the presence of black-hole graviton sinks. The implementation of the Boltzmann-Shannon entropy in the context of CQG-theory is met by expressing $S_{BS}$ as a 4−scalar functional of the invariant quantum probability density function ρ(s) given by Equation (6), namely

(7) $$S_{BS}\left(\rho (s)\right)=-\int_{U_{g}}d\left(g\right)\rho \left(s\right)\ln\rho \left(s\right).$$

A discussion on the physical meaning of the Boltzmann-Shannon entropy for the present research target is reported in Section 4.

Based on this identification, in the following, first a general analytical solution for the non-stationary quantum-gravity probability density function is obtained. Second, an analytical representation for the black-hole Boltzmann-Shannon entropy is determined for an invariant Gaussian profile of the quantum-gravity PDF. The choice of a Gaussian PDF ρ(s) is consistent with the analogous solution for the underlying probabilistic quantum-gravity wave function ψ(s) in the configuration space $U_{g}$. Under the prescription of physical requirements holding in statistical physics and information theory, the mathematical derivation of the Gaussian profile for the PDF $\rho \left(s\right)\in U_{g}$ is reported in Section 5. The profile of $S_{BS}\left(\rho (s)\right)$ is therefore associated with the quantum probability density ρ(s), which is normalized and summable in configuration space and conserved through quantum dynamics thanks to the Hamiltonian structure of the theory cast in conservative form.

The Gaussian PDF ρ(s) for the Boltzmann-Shannon entropy in CQG-theory is shown to depend on an arbitrary free invariant parameter (denoted as invariant thermal radius), which plays the role of the parameter β in kinetic statistics, namely the analog of the temperature in (2). The resolution method for the determination of such a parameter is not unique, but can be consistently addressed on a physical basis. As an example, in Section 6 the free parameter is determined as follows:

(1) By choosing the quantum-gravity nonlinear vacuum Bohm potential $V_{QM}\left(s\right)$ as the characteristic function expressing the quantum energy content of the solution under the condition of remaining defined, namely being non-vanishing, everywhere in the whole configuration domain. By construction, the potential $V_{QM}\left(s\right)$ is dimensionally normalized in terms of a free characteristic length-scale *L* to be properly prescribed according to the physical setting of the problem.
(2) By imposing the requirement that the mean value (i.e., the quantum expectation value) of the quantum-gravity nonlinear vacuum Bohm potential $V_{QM}\left(s\right)$ is proportional to the black-hole invariant-energy content, to be expressed through invariant relationships.
(3) By prescribing the characteristic length *L* of the vacuum Bohm potential in terms of a mass-dependent scale-length solution of the type L=L(M), where *M* is the black-hole mass.

This procedure permits characterizing the quantum-gravity PDF to the invariant properties of the black-hole identified with its mass *M*, energy and Schwarzschild radius through the relationship L=L(M). This framework is referred to as the L(M)−solution. The physical rationale behind this approach is discussed in Section 6.

Under these premises, a peculiar, i.e., non-trivial, functional dependence of the Boltzmann-Shannon entropy $S_{BS}\left(\rho (s)\right)$ on the black-hole surface area is shown to hold. For a Schwarzschild black-hole this is expressed in terms of the squared ratio

(8) $$S_{BS}\left(\rho (s)\right)\to S_{BS}\left({\left(\frac{A}{\ell_{P}^{2}}\right)}^{2}\right).$$

This profile is more complex than the linear relationship (1) and it might mark a point of difference with the Bekenstein-Hawking horizon entropy. In particular, the L(M)−solution can be given a physical meaning when it is interpreted in terms of information-statistic theory and the relationship with its semi-classical limit. In fact, as explained in Section 7, the Boltzmann-Shannon entropy $S_{BS}\left(\rho (s)\right)$ must be intended here as a quantum statistical entropy counting bulk quantum-gravity states accessible by the black-hole, rather than only horizon states. In this reference, the quadratic dependence should not be viewed as a contradiction with the semiclassical entropy area law (1) of $S_{BH}$ applying to so-called black-hole surface states. Rather, such a functional dependence can provide an explicit realization in agreement with the conjecture proved in Ref. [48] on the possibility of associating the entropy $S_{BS}\left(\rho (s)\right)$ to the accessible internal black-hole states, whose number is expected to be larger than that of surface states. On the same grounds, from both conceptual and formal points of view, the Boltzmann-Shannon entropy $S_{BS}\left(\rho (s)\right)$ of CQG-theory has not to be understood within holography theory or investigated through the language of holography principles. Hence, the same $S_{BS}\left(\rho (s)\right)$ should not necessarily be interpreted as a maximal holographic entropy bound [49], but rather as a microscopic informational measure associated with the quantum-gravity bulk state distribution.

The novel L(M)−solution remains further distinguished from the literature also because of the mathematical setting that is self-contained within quantum-gravity realm. This preserves manifest covariance and does not rely either on perturbative schemes, or on symmetry assumptions or the use of external test fields to extrapolate the entropic properties of black-holes. In the current formulation, in fact, thanks to the invariant character of CQG-theory, the quantum PDF ρ(s) as well as the entropy $S_{BS}\left(\rho (s)\right)$ are determined by effectively treating the black-hole as a fully quantum gravitational object by itself in its whole occupation domain. This excludes semi-classical or quantum-gravity approaches based on so-called Effective Field Theories [50,51,52], to be expressed by imposing quantum statistical behavior on the black-hole constituent matter. As an application, explicit analytically-tractable configurations are analyzed in Section 7, Subsection “Analytical Solution: Lower-Dimensional Case”, which which correspond to settings of lower-dimensional quantum-gravity excitation degrees of freedom with respect to the most general symmetric gravity tensor carrying ten independent non-vanishing components.

In order to shed further light on the problem, in Section 8 the subject is dedicated to the study of the comparison of quantum-gravity Boltzmann-Shannon theory with established literature treatments dealing with thermodynamic or kinetic-statistical entropies yielding the Bekenstein-Hawking black-hole surface entropy relation (1). In particular, it is analyzed under which mathematical and physical conditions the quantum-gravity PDF ρ(s) can generate a Boltzmann-Shannon entropy is able to reproduce the black-hole area law, and which logical implications follow from this requirement from the information-theory point of view.

Finally, in Section 9 an alternative configuration to the $L\left(M\right)-$solution is proposed. The aim is to prove that also within CQG-theory, physical regimes in which the black-hole entropy exhibits a functional dependence on the linear ratio $\frac{A}{\ell_{P}^{2}}$ represent possible meaningful solutions, even though with a different algebraic representation with respect to the Bekenstein-Hawking area law. The goal is met by imposing that the free scale-length *L* in the normalization of the vacuum Bohm potential is mass-independent and coincides with the Planck length $\ell_{P}$. The outcome obtained after imposing this condition is denoted as $\ell_{P}$-solution, to distinguish it from the $L\left(M\right)-$solution. From the physical point of view, the choice $L=\ell_{P}$ realizes a framework in which the quantum-gravity Boltzmann-Shannon entropy depends on the black-hole state only through its mass, and not in combination with its characteristic lenght (i.e., the Schwarzschild radius).

Concluding remarks and outlook perspectives are drawn in Section 10, while background notions on classical Lagrangian and Hamiltonian formulations of GR theory are reported in Appendix A and Appendix B, respectively.

## 2. Manifestly-Covariant Quantum Gravity Theory

In this section we proceed with the formulation of the manifestly-covariant quantum gravity theory (CQG-theory) appropriate for the study of black-hole entropic properties [34]. The mathematical framework and the formalism implemented in this section and subsequent ones arise from the manifestly-covariant synchronous Lagrangian and Hamiltonian formulations of GR theory recalled for completeness in Appendix A. In particular, the quantum setting adopted for CQG-theory is achieved in terms of an appropriate representation of the classical reduced Hamiltonian theory and the Hamiltonian structure $\left\{x_{R},H_{R}\right\}$ to be expressed in evolution form according to the details summarized in Appendix B. We notice however that the same target could be reached equivalently through the so-called Hamilton-Jacobi quantization starting from the Hamilton-Jacobi theory associated with the Hamiltonian theory, as proved in Ref. [53]. We refer similarly to Appendix A and Appendix B for the formal mathematical explanations of the notations adopted hereafter.

Before introducing the mathematical details of CQG-theory, for a better understanding of the framework, it is useful to summarize the following salient features of the theory and its main qualitative achievements. The distinguished features of CQG-theory with respect to alternative literature approaches are that:

(1) CQG-theory is consistent with the principle of manifest covariance and is established in terms of canonical quantization of a classical, regular, unconstrained and invertible Hamiltonian structure underlying classical GR [33,34].
(2) The quantum and classical Hamiltonian theories are formulated through Lagrangian and Hamiltonian variational principles. The classical Lagrangian description does not change the validity of the Hilbert-Einstein variational principle. Rather, it provides an alternative representation consistent with the DeDonder-Weyl symplectic formalism that is suitable for the subsequent Hamiltonian and canonical quantum representations.
(3) The classical Hamiltonian theory is consistent with the Einstein field equations, namely, the theory does not provide a modification of classical GR. It has been proved that the quantum gravity wave equation of CQG-theory reproduces correctly the Einstein field equations in the semiclassical limit [53].
(4) CQG-theory provides a quantum theory of gravitational fields cast in unitary conservative form, whereby quantum probability density and scalar products can be consistently defined in the corresponding Hilbert space, with the quantum-gravity wave-function evolving dynamically through a quantum-gravity wave equation [34].

Based on these premises, the following relevant outcomes have been established:

(1) CQG-theory is consistent with the emergent gravity phenomenon and the background independence, whereby the classical metric-tensor can be proved to be expressed consistently as a statistical average of underlying oscillatory quantum-gravity degrees of freedom [53,54].
(2) CQG-theory admits an indeterminacy principle and Heisenberg inequalities represented in terms of the standard deviations of the quantum-gravity tensor and its quantum conjugate momentum operator [55,56].
(3) CQG-theory admits the validity of an Ehrenfest theorem [57].
(4) CQG-theory allows treating space-time second-quantization effects and to determine a mathematical expression proving the quantum origin of cosmological constant [53].
(5) CQG-theory permits the display of the role of Planck length in classical and quantum Hamiltonian formulations of general relativity and to prove that Planck length emerges as the invariant quantum minimum effective length determined by the Heisenberg uncertainty principle of the same CQG setting [58,59].

We notice that some of the issues listed above represent still-unsolved conceptual problems of alternative approaches proposed in the literature as viable routes to the solution of the quantum-gravity problem or desirable conjectured features suggested in the same settings. In contrast, the present manifest-covariance formalism and the background Hamiltonian structure of the theory allow us to prove the validity of these properties in CQG-theory without affecting the foundational principles of classical GR and quantum mechanics.

Besides these achievements, other relevant issues still remain to be ascertained in order to complete the mathematical structure of CQG-theory, which represent future prospects for ongoing work. Among them, a crucial one is provided by the issue of renormalizability [60,61,62]. From a preliminary analysis, CQG-theory could provide a promising setting for its solution, yielding a quantization technique that might avoid the problem of renormalization altogether from its foundational character. In fact:

(1) CQG-theory is exact and non-perturbative, namely, it is not achieved as a perturbation of a classical background metric solution.
(2) CQG-theory does not require extra dimensions, but preserves the standard four dimensions of space-time. In contrast, it permits the introduction of additional degrees of freedom, corresponding to the quantum gravitational tensor, by means of adoption of superabundant Lagrangian variables that warrant consistency with DeDonder-Weyl formalism and the Hilbert-Einstein variational approach. This reflects in a novel identification of configuration space where quantum-gravity theory is developed.
(3) As a consequence, the Lagrangian variational principle does not provide modifications to the Hilbert-Einstein Lagrangian with the addition of higher-order derivative operators, as those which enter classical and quantum perturbative gravitational settings.
(4) The canonical state $x_{R}\left(s\right)\equiv \left(g\left(s\right),\pi \left(s\right)\right)$ of quantum gravitational tensor field and its conjugate momentum represent the set of quantum variables, whereby the tensor g(s) remains distinguished from the classical background metric tensor of space-time, the two being related however through emerging gravity phenomena. Interestingly, as pointed out below in the entropy derivation, the semiclassical limit of the quantum-gravity (Gaussian) wave function depends only on a single invariant free parameter of the theory.

These issues altogether pose a deeper question, namely, demanding to reformulate the full concept of renormalizability in the mathematical framework of CGQ-theory. In such a context, in fact, a more appropriate issue to prove should no longer pertain to a perturbative theory, but rather whether CQG-theory is ultraviolet-complete and therefore does not require any perturbative renormalization [63,64,65,66,67]. These conclusions, however, will be addressed in a separate future study, as they go beyond the scope of the present research.

In detail, the CQG-state is identified with a single 4−scalar complex function ψ(s) of the type

(9) $$\psi \left(s\right)\equiv \psi (g,\hat{g}\left(r\right),r\left(s\right),s),$$

which is denoted as CQG wave-function. This represents the wave function of a spin-2 quantum particle with invariant rest mass $m_{o}>0$, as warranted by the validity of the tensor product $\psi \left(s\right)={\hat{g}}_{\mu \nu }\psi^{\mu \nu }$. It is important to notice that, consistent with the underlying evolution-form representation of the Hamiltonian theory, ψ(s) is allowed to depend either explicitly on the proper-time *s* or implicitly through the Lagrangian variable $g\equiv \left\{g_{\mu \nu }\right\}\in$$U_{g}$, the background metric tensor $\hat{g}\left(r\left(s\right)\right)\equiv \left\{{\hat{g}}_{\mu \nu }\left(r\left(s\right)\right)\right\}$, and the Lagrangian path parametrization in terms of the geodesics $r\left(s\right)\equiv \left\{r^{\mu }\left(s\right)\right\}$. The function ψ defined by Equation (9) spans by construction a Hilbert space $\Gamma_{\psi }$ identified with a finite-dimensional linear vector space with associated scalar product. For arbitrary elements $\psi_{a,b}\left(s\right)\equiv \psi_{a,b}\left(g,\hat{g}\left(r\right),r\left(s\right),s\right)$ the latter is defined as

(10) $$\langle \psi_{a}|\psi_{b}\rangle \equiv \int_{U_{g}}d\left(g\right)\psi_{a}^{*}\left(g,\hat{g}\left(r\right),r\left(s\right),s\right)\psi_{b}\left(g,\hat{g}\left(r\right),r\left(s\right),s\right),$$

with $d\left(g\right)\equiv \prod_{\mu ,\nu =1\;\mathrm{to}\;4}$ $dg_{\mu \nu }$ being the canonical measure on $U_{g}$, while $\psi_{a}^{*}$ is the complex conjugate of $\psi_{a}$. This allows identifying the quantum probability density function (CQG-PDF) of $g\equiv \left\{g_{\mu \nu }\right\}$ associated with the CQG-state in the configuration space $U_{g}$ in terms of the real function $\rho \left(s\right)\equiv \rho (g,\hat{g}\left(r\right),r\left(s\right),s)$ expressed as

(11) $$\rho \left(s\right)\equiv {\left|\psi (s)\right|}^{2}.$$

A characteristic feature of this mathematical setting lies in the fact that the PDF retains the same physical meaning and probabilistic interpretation of non-relativistic quantum mechanics, for which the normalization condition for ρ(s) applies for consistency:

(12) $$\langle \psi |\psi \rangle \equiv \int_{U_{g}}d\left(g\right)\rho \left(s\right)=1.$$

The PDF ρ(s) introduced in this way has a tensorial character because it is represented by an invariant 4−scalar defined on the 4−dimensional space-time with respect to the background field ${\hat{g}}_{\mu \nu }$.

If $X\left(s\right)\equiv X(g,\hat{g}\left(r\right),r\left(s\right),s)$ denotes an arbitrary tensor function or a local tensor operator acting on an arbitrary wave-function $\psi \left(s\right)\in \Gamma_{\psi }$, the weighted integral

(13) $$\langle \psi |X\psi \rangle \equiv \int_{U_{g}}d\left(g\right)\psi^{*}\left(s\right)X\left(s\right)\psi \left(s\right)$$

identifies the CQG-expectation value of *X*. Then, by denoting with $X^{*}\left(s\right)$ the complex conjugate of X(s), if

(14) $$\langle \psi |X\psi \rangle =\langle X^{*}\psi |\psi \rangle \equiv \int_{U_{g}}d\left(g\right)\psi \left(s\right)X^{*}\left(s\right)\psi^{*}\left(s\right),$$

it means that $\langle \psi |X\psi \rangle$ is real and as a consequence *X* identifies a CQG-observable. An example of this type is provided by the 4−tensor function $X\equiv g_{\mu \nu }$, for which

(15) $$\langle \psi |g_{\mu \nu }\psi \rangle =\int_{U_{g}}d\left(g\right)g_{\mu \nu }\rho \left(g,\hat{g}\left(r\right),r\left(s\right),s\right)={\tilde{g}}_{\mu \nu }\left(\hat{g}\left(r\right),r\left(s\right),s\right)$$

represents the corresponding CQG-expectation value at $r\equiv r\left(s\right)\in \left(Q^{4},\hat{g}\left(r\right)\right)$. Another relevant example is given by the CQG-expectation value of the momentum operator $\pi^{(q)\mu \nu }\equiv -i\hbar \frac{\partial }{\partial g_{\mu \nu }}$, defined by the integral

(16) $$\begin{array}{ccc} \langle \psi |\pi^{(q)\mu \nu }\psi \rangle & = & \int_{U_{g}}d\left(g\right)\psi \left(s\right)\left(-i\hbar \frac{\partial }{\partial g_{\mu \nu }}\right)\psi^{*}\left(s\right)\\ & \equiv & {\tilde{\pi }}^{(q)\mu \nu }\left(\hat{g}\left(r\right),r\left(s\right),s\right), \end{array}$$

where ${\tilde{\pi }}^{(q)\mu \nu }$ is a real tensor field.

The quantum Hamiltonian operator is obtained from the prescription of the classical Hamiltonian $H_{R}$ [57]. In order to have canonical momenta with the dimension of an action as is customary in quantum mechanics, a dimensional normalization of the Hamiltonian structure is introduced through the map $\left\{x_{R},H_{R}\right\}\to \left\{{\bar{x}}_{R},{\bar{H}}_{R}\right\}$. This produces the dimensionally normalized classical Hamiltonian structure $\left\{{\bar{x}}_{R},{\bar{H}}_{R}\right\}$. Here, the canonical state ${\bar{x}}_{R}\equiv \left\{{\bar{g}}_{\mu \nu },{\bar{\pi }}_{\mu \nu }\right\}$ is such that ${\bar{g}}_{\mu \nu }\equiv g_{\mu \nu }$, while ${\bar{\pi }}_{\mu \nu }=\frac{\alpha L}{k}\pi_{\mu \nu }$ is the normalized conjugate momentum, where $\kappa =\frac{c^{3}}{16\pi G}$, *L* is a 4−scalar scale length and α is a suitable dimensional 4−scalar, both defined in Ref. [34]. In addition, the real 4−scalar Hamiltonian field ${\bar{H}}_{R}$ is defined as

(17) $${\bar{H}}_{R}\left({\bar{x}}_{R},\hat{g},r,s\right)={\bar{T}}_{R}\left(\bar{g},\hat{g},r,s\right)+\bar{V}\left(\bar{g},\hat{g},r,s\right),$$

with ${\bar{T}}_{R}\left(\bar{g},\hat{g},r,s\right)\equiv \frac{{\bar{\pi }}^{\mu \nu }{\bar{\pi }}_{\mu \nu }}{2\alpha L}$ and $\bar{V}\left(\bar{g},\hat{g},r,s\right)\equiv h\alpha L\left[g^{\mu \nu }{\hat{R}}_{\mu \nu }-2\Lambda \right]$ being the normalized effective kinetic and potential densities. The canonical quantization rules for CQG-theory are then exemplified by the CQG-correspondence principle, to be realized by the map

(18) $$\left\{\begin{array}{c} {\bar{g}}_{\mu \nu }\equiv g_{\mu \nu }\to g_{\mu \nu }^{\left(q\right)}\equiv g_{\mu \nu },\\ {\bar{\pi }}_{\mu \nu }\to \pi_{\mu \nu }^{\left(q\right)}\equiv -i\hbar \frac{\partial }{\partial g^{\mu \nu }},\\ {\bar{H}}_{R}\to {\bar{H}}_{R}^{\left(q\right)}={\bar{T}}_{R}^{\left(q\right)}\left(\bar{\pi }\right)+\bar{V}. \end{array}\right.$$

Here, ${\bar{H}}_{R}^{\left(q\right)}$ is the Hermitian CQG-Hamiltonian operator, $x^{\left(q\right)}\equiv \left\{g_{\mu \nu }^{\left(q\right)},\pi_{\mu \nu }^{\left(q\right)}\right\}$ is the quantum canonical state and $\pi_{\mu \nu }^{\left(q\right)}$ is the quantum momentum operator prescribed so that the commutator $\left[g_{\mu \nu }^{\left(q\right)},\pi^{(q)\alpha \beta }\right]=i\hbar \delta_{\mu }^{\alpha }\delta_{\nu }^{\beta }$ holds, while ${\bar{T}}_{R}^{\left(q\right)}\left(\bar{\pi }\right)=\frac{\pi^{(q)\mu \nu }\pi_{\mu \nu }^{\left(q\right)}}{2\alpha L}$ is the kinetic quantum operator.

Finally, collecting these results, the CQG-quantum wave equation that advances in proper-time the quantum state ψ is obtained by promoting the Poisson brackets to quantum commutator yielding

(19) $$i\hbar \frac{\partial }{\partial s}\psi \left(s\right)+\left[\psi \left(s\right),{\bar{H}}_{R}^{\left(q\right)}\right]=0,$$

where $\left[\psi \left(s\right),{\bar{H}}_{R}^{\left(q\right)}\right]\equiv -{\bar{H}}_{R}^{\left(q\right)}\psi \left(s\right)$. The CQG-wave Equation (19) describes the dynamical evolution of the quantum state ψ(s) along the geodetics of the background metric tensor ${\hat{g}}_{\mu \nu }\left(r\right)$. Remarkably, the same Equation (19) is realized by a first order partial differential equation, which must be supplemented by initial conditions prescribing for all $r\left(s_{o}\right)=r_{o}\in \left(Q^{4},\hat{g}\left(r\right)\right)$ the function $\psi \left(s_{o}\right)=\psi_{o}\left(g,\hat{g}\left(r_{o}\right),r_{o}\right)$, as well as the limiting boundary conditions $\lim_{g\to \infty }\psi \left(g,\hat{g}\left(r\right),r\left(s\right),s\right)=0$ on the improper boundary of configuration space $U_{g}$.

## 3. The Quantum Hydrodynamic Equations

The CQG-quantum wave equation provided by Equation (19) is equivalent to a set of quantum hydrodynamic equations (QHE) to be identified respectively with the continuity equation for the quantum PDF $\rho \left(s\right)=\rho (g,\hat{g}\left(r\right),r\left(s\right),s)$ and the Hamilton-Jacobi equation for the quantum phase-function $S^{\left(q\right)}\left(s\right)=S^{\left(q\right)}\left(g,\hat{g}\left(r\right),r\left(s\right),s\right)$. The result can be reached by expressing the complex function ψ(s) in terms of the exponential Madelung representation as

(20) $$\psi \left(s\right)=\sqrt{\rho (s)}\exp\left\{\frac{i}{\hbar }S^{\left(q\right)}\left(s\right)\right\},$$

where ρ(s) and $S^{\left(q\right)}\left(s\right)$ are real 4−scalar field functions. By replacing Equation (20) in the CQG-wave Equation (19) one then obtains the following set of real PDEs written in Eulerian form and advancing in proper-time, respectively, ρ(s) and $S^{\left(q\right)}\left(s\right)$:

(21) $$\begin{array}{ccc} \frac{\partial \rho (s)}{\partial s}+\frac{\partial }{\partial g_{\mu \nu }}\left(\rho \left(s\right)V_{\mu \nu }\left(s\right)\right) & = & 0, \end{array}$$

(22) $$\begin{array}{ccc} \frac{\partial S^{\left(q\right)}\left(s\right)}{\partial s}+{\bar{H}}_{c}^{\left(q\right)} & = & 0. \end{array}$$

These are referred to as CQG-quantum continuity and CQG-quantum Hamilton-Jacobi equations, here expressed in conservative form, namely warranting conservation of quantum probability. The mathematical proof concerning the derivation of the QHE (21) and (22) can be found in Ref. [53]. Concerning the notation, the quantum hydrodynamics fields $\rho \left(s\right)\equiv \rho \left(g,\hat{g},s\right)$ and $S^{\left(q\right)}\left(s\right)\equiv S^{\left(q\right)}\left(g,\hat{g}.s\right)$ are assumed to depend smoothly on the tensor field $g\equiv \left\{g_{\mu \nu }\right\}\in U_{g}$ and to admit a Lagrangian path parametrization in terms of the geodetics $r\left(s\right)\equiv \left\{r^{\mu }\left(s\right)\right\}$ associated with the background tensor field $\hat{g}\left(r\right)\equiv \left\{{\hat{g}}_{\mu \nu }\left(r\right)\right\}$. Furthermore, $V_{\mu \nu }\left(s\right)\equiv \frac{1}{\alpha L}\frac{\partial S^{\left(q\right)}}{\partial g^{\mu \nu }}$, while ${\bar{H}}_{c}^{\left(q\right)}$ is the effective quantum Hamiltonian density

(23) $${\bar{H}}_{c}^{\left(q\right)}=\frac{1}{2\alpha L}\frac{\partial S^{\left(q\right)}}{\partial g^{\mu \nu }}\frac{\partial S^{\left(q\right)}}{\partial g_{\mu \nu }}+V_{QM}+V.$$

In the previous expression, $V\equiv V(g,\hat{g}\left(r\right),r,s)$ represents the effective potential density and $V_{QM}\equiv V_{QM}\left(s\right)=V_{QM}\left(g,\hat{g}\left(r\right),r,s\right)$ is the so-called Bohm-like effective quantum potential [68,69,70] which is prescribed as

(24) $$V_{QM}\left(s\right)=\frac{\hbar^{2}}{8\alpha L}\frac{\partial \ln\rho \left(s\right)}{\partial g^{\mu \nu }}\frac{\partial \ln\rho \left(s\right)}{\partial g_{\mu \nu }}-\frac{\hbar^{2}}{4\alpha L}\frac{\partial^{2}\rho \left(s\right)}{\rho \left(s\right)\partial g_{\mu \nu }\partial g^{\mu \nu }}.$$

This potential contribution arises due to the non-uniformity of the quantum PDF in configuration space, namely because generally $\frac{\partial }{\partial g_{\mu \nu }}\rho \left(s\right)\neq 0$.

Also QHE can be cast in a trajectory-based (Lagrangian path) representation, which permits the establishment of a Bohmian interpretation of the quantum theory. In the present context, this requires the introduction (for all s∈I) of the correspondence

(25) $$s\to g_{L}\left(s\right)\equiv g_{L}\left(r\left(s\right),s\right),$$

so that any $g_{\mu \nu }\equiv g_{L\mu \nu }\left(s\right)\in U_{g}$, to be denoted as Lagrangian path, represents a curve belonging to the ensemble $\left\{g_{L}\left(s\right),\forall s\in I\right\}$ of the configuration space $U_{g}$, to be identified with a characteristics associated with the tensor “velocity” field $V_{\mu \nu }\left(g,r,s\right)$. Hence, any $g_{L\mu \nu }\left(s\right)$ fulfills the initial-value problem

(26) $$\left\{\begin{array}{c} \frac{D}{Ds}g_{L\mu \nu }\left(s\right)=V_{\mu \nu }\left(g_{L}\left(s\right),s\right),\\ g_{\mu \nu }\left(s_{o}\right)=g_{\mu \nu }^{\left(o\right)}, \end{array}\right.$$

where $\frac{D}{Ds}$ identifies the covariant Lagrangian-path derivative defined as the operator

(27) $$\frac{D}{Ds}\equiv {\left.\frac{d}{ds}\right|}_{g_{L\mu \nu }\left(s\right)}+V_{\mu \nu }\left(g_{L}\left(s\right),s\right)\frac{\partial }{\partial g_{L\mu \nu }},$$

where

(28) $${\left.\frac{d}{ds}\right|}_{g_{L\mu \nu }\left(s\right)}\equiv {\left.\frac{D}{Ds}\right|}_{g_{\mu \nu }}={\left[{\left.\frac{\partial }{\partial s}\right|}_{r}+t^{\alpha }\nabla_{\alpha }\right]}_{g_{L\mu \nu }},$$

is the covariant s−derivative performed at constant $g_{L\mu \nu }\equiv g_{L\mu \nu }\left(s\right)$, while the second term on the rhs of Equation (27) is the convective derivative performed with respect to the Lagrangian coordinates $g_{L\mu \nu }\left(s\right)$ while keeping constant $\hat{g}\left(r\right)\equiv \left\{{\hat{g}}_{\mu \nu }\left(r\left(s\right)\right)\right\}$.

From the validity of Equation (26), by integration one obtains the formal solution for the Lagrangian-path trajectory $\left\{g_{L}\left(s\right),\forall s\in I\right\}$, namely

(29) $$g_{L\mu \nu }\left(s\right)=g_{\mu \nu }^{\left(o\right)}+\int_{s_{o}}^{s}ds^{\prime }V_{\mu \nu }\left(g_{L}\left(s^{\prime }\right),s^{\prime }\right).$$

The uniqueness of the solution $g_{L\mu \nu }\left(s\right)$ given by Equation (29) is warranted by the additional prescription of the initial condition $g_{\mu \nu }^{\left(o\right)}$. It means that the mapping

(30) $$g_{L\mu \nu }\left(s_{o}\right)=g_{\mu \nu }^{\left(o\right)}\Leftrightarrow g_{L\mu \nu }\left(s\right)$$

identifies a classical dynamical system, namely a diffeomorphism mapping in each other two arbitrary points $g_{L\mu \nu }\left(s_{o}\right)$ and $g_{L\mu \nu }\left(s\right)$ belonging to the same Lagrangian path. As a consequence, the Liouville theorem warrants that the Jacobian determinant of the transformation (30) is provided by

(31) $$\left|\frac{\partial g_{L}\left(s\right)}{\partial g_{L}\left(s_{o}\right)}\right|=\exp\left\{\int_{s_{o}}^{s}ds^{\prime }\frac{\partial V_{\mu \nu }\left(g_{L}\left(s^{\prime }\right),s^{\prime }\right)}{\partial g_{L\mu \nu }\left(s^{\prime }\right)}\right\}.$$

The Lagrangian-path representation of CQG-theory requires the parametrization of the quantum fields in terms of $g_{L\mu \nu }\left(s\right)$ by means the formal replacement $g\to g_{L}\left(s\right)$, e.g., for the quantum state letting $\psi \equiv \psi (g_{L}\left(s\right),s)$, and similarly for the quantum fluid fields

(32) $$\left\{\rho ,S^{\left(q\right)}\right\}\equiv \left\{\rho \left(g_{L}\left(s\right),s\right),S^{\left(q\right)}\left(g_{L}\left(s\right),s\right)\right\}.$$

Then, in terms of the tensor velocity field $V_{\mu \nu }\left(g_{L}\left(s\right),s\right)\equiv \frac{1}{\alpha L}\frac{\partial S^{\left(q\right)}\left(g_{L}\left(s\right),r\left(s\right),s\right)}{\partial g_{L}^{\mu \nu }\left(s\right)}$, the corresponding Lagrangian-path representation of the quantum hydrodynamic Equations (21) and (22) becomes

(33) $$\left\{\begin{array}{c} \frac{D}{Ds}\rho \left(g_{L}\left(s\right),s\right)=-\rho \left(g_{L}\left(s\right),s\right)\frac{\partial V_{\mu \nu }\left(g_{L}\left(s\right),s\right)}{\partial g_{L\mu \nu }\left(s\right)},\\ \frac{D}{Ds}S^{\left(q\right)}\left(g_{L}\left(s\right),s\right)=V_{\mu \nu }\left(g_{L}\left(s\right),s\right)\frac{\partial S^{\left(q\right)}\left(g_{L}\left(s\right),s\right)}{\partial g_{L\mu \nu }\left(s\right)}\\ -{\bar{H}}_{c}^{\left(q\right)}\left(g_{L}\left(s\right),s\right), \end{array}\right.$$

where similarly ${\bar{H}}_{c}^{\left(q\right)}\left(g_{L}\left(s\right),s\right)$ is the $g_{L}\left(s\right)$-parametrization of the effective quantum Hamiltonian density (23). In particular, the first equation in (33) can be formally integrated to give the Lagrangian-path parametrized integral continuity equation

(34) $$\rho \left(g_{L}\left(s\right),s\right)=\rho \left(g_{L}\left(s_{o}\right),s_{o}\right)\exp\left\{-\int_{s_{o}}^{s}ds^{\prime }\frac{\partial V_{\mu \nu }\left(g_{L}\left(s^{\prime }\right),s^{\prime }\right)}{\partial g_{L\mu \nu }\left(s^{\prime }\right)}\right\},$$

with $\rho \left(g_{L}\left(s_{o}\right),s_{o}\right)\equiv \rho \left(g_{L}\left(s_{o}\right),r\left(s_{o}\right)=r_{o},s_{o}\right)$ denoting the initial quantum PDF, namely

(35) $$\rho \left(g_{L}\left(s_{o}\right),r\left(s_{o}\right)=r_{o},s_{o}\right)=\rho_{o}\left(g_{L}\left(s_{o}\right),r_{o}\right).$$

Invoking also the Liouville theorem (31), the following conservation laws are obtained:

(36) $$d\left(g_{L}\left(s\right)\right)\rho \left(g_{L}\left(s\right),s\right)=d\left(g_{L}\left(s_{o}\right)\right)\rho \left(g_{L}\left(s_{o}\right),s_{o}\right),$$

(37) $$\int_{U_{g}}d\left(g_{L}\left(s\right)\right)\rho \left(g_{L}\left(s\right),s\right)=\int_{U_{g}}d\left(g_{L}\left(s_{o}\right)\right)\rho \left(g_{L}\left(s_{o}\right),s_{o}\right)=1,$$

which proves the consistency of the theory with the quantum unitarity principle.

As a final remark, the Lagrangian-path formalism developed here allows to define, in terms of the PDF $\rho (g_{L}\left(s\right),s)$, the quantum expectation value of arbitrary observables that are identified with ordinary tensor functions. To illustrate the issue, let us consider a generic observable represented by an ordinary s−dependent real function $X\left(s\right)\equiv X(g_{L}\left(s\right),s)$. Then, by construction, its quantum expectation value is given by the configuration-space integral

(38) $$\langle X(s)\rangle =\int_{U_{g}}d\left(g_{L}\left(s\right)\right)\rho \left(g_{L}\left(s\right),s\right)X\left(g_{L}\left(s\right),s\right),$$

where the integration is performed in terms of $\rho (g_{L}\left(s\right),s)$ with respect to $g_{L}\left(s\right)$, keeping constant the background metric tensor $\hat{g}\left(r\right)\equiv$$\hat{g}\left(r\left(s\right)\right)$.

## 4. CQG Boltzmann-Shannon Entropy

In this section, the definition of the notion of quantum entropy for the quantum gravitational field of CQG-theory is established. The conceptual framework follows the well-known approach developed in the context of non-relativistic QM, where the notion of quantum statistical entropy was first developed (for a related discussion, see, for example, Ref. [53]). However, the same approach can in principle be formulated for an arbitrary quantum theory, which is endowed with a corresponding quantum PDF ρ defined on a configuration space (*D*) and satisfying a quantum hydrodynamic continuity equation.

We start by introducing the quantum entropy density, i.e., the partition function, which is identified with the 4−scalar quantum phase-function

(39) $$E_{BS}\left(\rho \right)\equiv -\ln\rho ,$$

with ρ=ρ(g,s) being the quantum PDF defined through Equation (20) and determined explicitly by the integral solution (34). This prescription coincides formally with that of quantum differential entropy adopted in quantum field theory and quantum mechanics (see, for example, Ref. [53]). As such, $E_{BS}\left(\rho \right)$ represents a real 4−scalar field, which is locally defined everywhere in the background space-time $\left\{Q^{4},\hat{g}\right\}$ and may depend also explicitly on proper time *s*.

Then, we can introduce the notion of quantum differential entropy, which coincides here with the quantum expectation value

(40) $$S_{BS}\left(\rho \right)\equiv \langle E_{BS}\left(\rho \right)\rangle ,$$

with $E_{BS}\left(\rho \right)$ being the quantum phase function (39). The expectation value $\langle E_{BS}\left(\rho \right)\rangle$ is defined here as the integral performed on the configuration space *D* and weighted with respect to the corresponding quantum PDF ρ. Hence, it is actually realized by the corresponding quantum Boltzmann-Shannon entropy (or equivalently known as information-theory statistical Shannon entropy) for the same PDF, namely

(41) $$\langle E_{BS}\left(\rho \right)\rangle =\int_{D}d\Omega \rho E_{BS}\left(\rho \right),$$

where dΩ is the configuration-space volume element. From the physical point of view, $S_{BS}\left(\rho \right)$ is related to the ignorance in the form of the quantum PDF ρ, i.e., in turn, a measure of the ignorance of the knowledge of the corresponding quantum state ψ. The notion of quantum entropy adopted here is not a thermodynamical one, but rather the same as that used in the statistical formulation of information theory, thus bringing a challenging picture in application to black-hole entropic properties.

It is worth commenting on the physical basis behind the choice of the Boltzmann-Shannon entropy. The Boltzmann-Shannon entropy can be viewed as a generalization of the statistical entropy $S_{k}$ that yields the thermodynamic Bekenstein-Hawking entropy $S_{BH}$. In fact, from the physical point of view, the Boltzmann-Shannon entropy is a microscopic state-counting entropy that applies when the states associated with the partition function are not equiprobable. Accordingly, $S_{BS}$ represents the mean value (i.e., the quantum expectation value) of the partition function weighted over the same PDF for non-equiprobable states. However, a crucial aspect that marks a distinction between the Boltzmann-Shannon entropy adopted here and the thermodynamics Bekenstein-Hawking entropy lies in the choice of the statistics. In fact, for thermodynamic approaches, the latter setting relies on phase-space kinetic statistics with the corresponding probability density being defined in the particle momentum space (e.g., the Boltzmann or the Bose-Einstein statistics). In the current setting, instead, the statistics are the intrinsic ones associated with the quantum PDF generated by the Madelung formalism for the representation of the wave function. The quantum-gravity PDF is then defined on the configuration space $U_{g}$ of quantum tensors $g_{\mu \nu }$, and similarly the quantum-average expectation values (like the one defining the entropic integral) are performed on the same configuration space. This kind of quantum statistics is expected to yield additional or complementary information about black-hole entropic properties with respect to phase-space kinetic statistics.

The Boltzmann-Shannon entropy $S_{BS}$ has formal similarities with the von Neumann entropy $S_{vN}$ that describes the statistical uncertainty of quantum systems and is regarded as the quantum counterpart of the Shannon entropy applied in classical information theory. In fact, both $S_{BS}$ implemented here and $S_{vN}$ are rooted in the intrinsic quantum statistics attached to the quantum state and do not rely on phase-space kinetic statistics. The von Neumann entropy is defined as the trace $S_{vN}=-$tr(ρlnρ), where ρ is the density matrix of a quantum-mechanical system. For this reason, its adoption is appropriate when the system admits a discrete spectrum of quantum eigenstates. On the contrary, $S_{BS}$ should be viewed as a continuous-information entropy, which is appropriate for the quantum-gravity problem of interest here under the adoption of Madelung formalism when Equations (6) and (20) apply. In addition, its representation in terms of a 4−scalar warrants validity of manifest covariance.

In the framework of CQG-theory, the configuration space *D* is identified with the *g*-space $U_{g}$ of quantum tensors $g_{\mu \nu }$, with $d\left(g\right)\equiv \prod_{\mu ,\nu =1\;\mathrm{to}\;4}$ $dg_{\mu \nu }$ being the canonical measure on $U_{g}$, while the quantum PDF ρ is represented as $\rho (g_{L}\left(s\right),s)$ according to Equation (34), so that the entropy functional takes the form

(42) $$\begin{array}{ccc} S_{BS}\left(\rho (g_{L}\left(s\right),s)\right) & = & \langle E_{BS}\left(g_{L}\left(s\right),s\right)\rangle \\ & = & \int_{U_{g}}d\left(g\right)\rho \left(g_{L}\left(s\right),s\right)E_{BS}\left(g_{L}\left(s\right),s\right), \end{array}$$

to be denoted as CQG Boltzmann-Shannon entropy. Substituting the explicit expression for $E_{BS}\left(g_{L}\left(s\right),s\right)$ from Equation (39) then yields

(43) $$S_{BS}\left(\rho (g_{L}\left(s\right),s)\right)=-\int_{U_{g}}d\left(g\right)\rho \left(g_{L}\left(s\right),s\right)\ln\rho \left(g_{L}\left(s\right),s\right).$$

The corresponding initial quantum Boltzmann-Shannon entropy is obtained by evaluating the previous integral for the initial state at $s=s_{o}$, yielding

(44) $$S_{BS}\left(\rho (g_{L}\left(s_{o}\right),s_{o})\right)=-\int_{U_{g}}d\left(g\right)\rho \left(g_{L}\left(s_{o}\right),s_{o}\right)\ln\rho \left(g_{L}\left(s_{o}\right),s_{o}\right).$$

The remarkable features of the present formalism are the following ones:

(1) There exists a Hamiltonian quantum gravity theory yielding a corresponding set of quantum hydrodynamic equations evolving in proper-time the quantum fluid fields. These hydrodynamic equations admit a representation both in Eulerian and Lagrangian forms. In this reference, the notion of Boltzmann-Shannon entropy appears to be of very general validity, and not only associated with black-hole physics. It arises as a property of CQG-theroy, characteristic of the quantum gravity state because of non-vanishing quantum PDF.
(2) The quantum PDF satisfies a continuity equation that warrants the unitary principle and the conservation of quantum probability associated with the quantum wave function.
(3) The quantum PDF as well as all other functions expressed by it, and in particular the quantum Boltzmann-Shannon entropy, are 4−scalars, consistent with the principle of manifest covariance.
(4) The quantum PDF evolves in proper time according to the continuity equation and the solution of the momentum equation. An important aspect is represented by the prescription of the initial quantum PDF, which remains unaltered in the stationary case, or it evolves quantum-dynamically according to QHE. The dynamical evolution equation of the PDF is then transferred to the Boltzmann-Shannon entropy. The connection with thermodynamic entropy and black-hole entropy law pertains to the initial or, equivalently, the stationary PDF.

## 5. Prescription of Quantum Gravity PDF

From the previous considerations, it follows that the physical characterization of the solution to concrete systems or space-time mathematical settings can be achieved in two steps:

(1) First, through an appropriate prescription of the initial quantum gravity PDF $\rho (g_{L}\left(s_{o}\right),s_{o})$ which is required for the integration of the CQG continuity equation in Lagrangian form.
(2) Second, in case of non-stationary configurations (in the sense of proper-time *s*), through the quantum dynamical evolution determined by the simultaneous validity of the coupled QHE for the quantum fields $\left\{\rho ,S^{\left(q\right)}\right\}$ subject to the appropriate prescription of the quantum-gravity Hamiltonian operator for the case of interest.

Incidentally, in the particular case of a stationary configuration, the initial PDF does not evolve, and it coincides with the solution independent of proper-time *s*. We also notice that necessarily the knowledge of the quantum-gravity PDF permits the evaluation of the corresponding Boltzmann-Shannon entropy at any generic proper-time *s* according to Equation (43).

Let us therefore consider the realization and the corresponding representation for $\rho (g_{L}\left(s_{o}\right),s_{o})$. The quantum statistics in such a case is developed in the configuration space $U_{g}$ of quantum tensors $g_{\mu \nu }$ where canonical momenta become quantum operators, according to the standard formalism of quantum mechanics. The same configuration space $U_{g}$ must be distinguished from the background space-time ${\hat{g}}_{\mu \nu }$ of physical observables. Although the form of $\rho (g_{L}\left(s_{o}\right),s_{o})$ may not be unique, one can nevertheless impose stringent conditions constraining its solution. In particular, following the analogous treatment given in Ref. [53], in order to determine the form of the quantum PDF $\rho (g_{L}\left(s_{o}\right),s_{o})$ the following requirements are imposed:

(1) The PDF must be defined and summable, namely, integrable, in the whole configuration space $U_{g}$ of quantum tensors $g_{\mu \nu }$ in order to warrant, among others, the normalization of quantum probability density as well as the mathematical consistency of the integral definition of entropy given by Equation (42).
(2) Additionally, as customary in statistical physics and information theory, the prescription of the initial PDF can be obtained by invoking the so-called Principle of Entropy Maximization [71]. This amounts to maximizing the Boltzmann-Shannon entropy associated with the same PDF and subject to appropriate constraints for its statistical moments [53]. The latter includes the normalization condition of the PDF, the representation of its mean tensor, which determines the average value of the distribution and its shift properties in configuration space $U_{g}$, and the so-called thermal width, namely the spread of the distribution around its mean value.

Combining these physical and mathematical requirements, one finds that in the whole configuration domain $U_{g}$ the initial PDF $\rho (g_{L}\left(s_{o}\right),s_{o})$ is represented in exponential form as

(45) $$\rho \left(g_{L}\left(s_{o}\right),s_{o}\right)=\frac{1}{\pi^{5}r_{th}^{10}}\exp\left\{-\frac{{\left(g\right)}^{2}}{r_{th}^{2}}\right\}\equiv \rho_{G}\left(g_{L}\left(s_{o}\right),s_{o}\right),$$

with $\rho_{G}\left(g_{L}\left(s_{o}\right),s_{o}\right)$ denoting a Gaussian PDF in which both $r_{th}^{2}$ and ${\left(g\right)}^{2}$ are 4−scalars. In particular, $r_{th}^{2}$ is an invariant constant independent of (r,s), which is written as a squared quantity to stress that it must be $r_{th}^{2}>0$. Instead, the 4−scalar ${\left(g\right)}^{2}$ is defined through the tensor product as

(46) $${\left(g\right)}^{2}\equiv g_{\mu \nu }g^{\mu \nu }.$$

Then, if we denote with $\rho_{G}\left(g_{L}\left(s\right),s\right)$ the evolved Gaussian PDF (45) evaluated for a generic 4−position r(s) generally different from the initial one $r_{o}\equiv r\left(s_{o}\right)$, it is possible to show that a formal solution ρ(g) of the quantum continuity equation can be taken of the form

(47) $$\rho_{G}\left(g_{L}\left(s\right),s\right)=\rho_{G}\left(g_{L}\left(s_{o}\right),s_{o}\right)\exp\left\{-\int_{s_{o}}^{s}ds^{\prime }\frac{\partial V_{\mu \nu }\left(g_{L}\left(s^{\prime }\right),s^{\prime }\right)}{\partial g_{L\mu \nu }\left(s^{\prime }\right)}\right\}.$$

We notice that, according to requirement 2 above, an alternative, albeit equivalent, choice for the quantum-gravity PDF is represented by the shifted Gaussian function

(48) $$\rho_{G-\hat{g}}\left(g_{L}\left(s_{o}\right),s_{o}\right)=\frac{1}{\pi^{5}r_{th}^{10}}\exp\left\{-\frac{{\left(g-\hat{g}\right)}^{2}}{r_{th}^{2}}\right\},$$

where ${\left(g-\hat{g}\right)}^{2}\equiv \left(g_{\mu \nu }-{\hat{g}}_{\mu \nu }\right)\left(g^{\mu \nu }-{\hat{g}}^{\mu \nu }\right)$. The difference is that here $\rho_{G-\hat{g}}\left(g_{L}\left(s_{o}\right),s_{o}\right)$ is a Gaussian centered around the background metric-tensor solution ${\hat{g}}_{\mu \nu }$. This kind of representation was proved to be consistent with the phenomenon of emergent gravity, namely the fact that the background metric tensor arises consistently as the quantum expectation value of the quantum-gravity tensor PDF [53]. In fact, thanks to the symmetry of the functional, the mean value $\langle g_{\mu \nu }\rangle$ yields identically

(49) $$\langle g_{\mu \nu }\rangle =\int_{U_{g}}d\left(g\right)g_{\mu \nu }\rho_{G-\hat{g}}\left(g_{L}\left(s_{o}\right),s_{o}\right)={\hat{g}}_{\mu \nu }.$$

Hence, the quantum-gravity PDF generates the background space-time solution as emerging from quantum gravitational states oscillating around the mean value $\langle g_{\mu \nu }\rangle$. The two PDFs $\rho_{G}\left(g_{L}\left(s_{o}\right),s_{o}\right)$ and $\rho_{G-\hat{g}}\left(g_{L}\left(s_{o}\right),s_{o}\right)$ are related by a shift in configuration space $U_{g}$ determining the symmetry point around which the Gaussian is centered. For this reason, for simplicity of analytical calculations but without loss of generality, in the following we shall use the Gaussian PDF $\rho_{G}\left(g_{L}\left(s_{o}\right),s_{o}\right)$ to study the entropic properties of black-holes.

Let us now consider the calculation of two relevant quantities of the PDF $\rho_{G}\left(g_{L}\left(s_{o}\right),s_{o}\right)$, to be identified with suitable moments of the same distribution.

The first one is the proof of the normalization coefficient $\frac{1}{\pi^{5}r_{th}^{10}}$ in Equation (45). To this aim, let us write the PDF as

(50) $$\rho_{G}\left(g_{L}\left(s_{o}\right),s_{o}\right)=\frac{1}{N}\exp\left\{-\frac{{\left(g\right)}^{2}}{r_{th}^{2}}\right\},$$

and let us impose the normalization condition

(51) $$\int_{U_{g}}d\left(g\right)\rho_{G}\left(g_{L}\left(s_{o}\right),s_{o}\right)=1,$$

which is equivalent to

(52) $$\int_{U_{g}}d\left(g\right)\exp\left\{-\frac{{\left(g\right)}^{2}}{r_{th}^{2}}\right\}=N,$$

where the integral must be performed over the g−space composed of ten independent components of the symmetric tensor $g_{\mu \nu }$. In detail, writing for convenience of notation $\rho_{G}\left(g_{L}\left(s_{o}\right),s_{o}\right)\equiv \rho_{G}\left(s_{o}\right)$, the integral for the ij−th components is written as

(53) $$\int_{U_{g}}d\left(g\right)\to \underset{ij}{\Pi }\int_{-\infty }^{+\infty }dg_{\left(i\right)\left(j\right)},$$

where *i* and *j* identify the ten independent components of the symmetric tensor $g_{\mu \nu }$. Invoking the symmetry of the integral we have that

(54) $$\begin{array}{ccc} N & = & \int_{U_{g}}d\left(g\right)\exp\left\{-\frac{{\left(g\right)}^{2}}{r_{th}^{2}}\right\}\\ & = & \underset{ij}{\Pi }\int_{-\infty }^{+\infty }dg_{\left(i\right)\left(j\right)}\exp\left\{-\frac{{\left(g\right)}^{2}}{r_{th}^{2}}\right\}\\ & = & \underset{ij}{\Pi }\left[2\int_{0}^{+\infty }dg_{\left(i\right)\left(j\right)}\exp\left\{-\frac{{\left(g\right)}^{2}}{r_{th}^{2}}\right\}\right]\\ & = & \underset{ij}{\Pi }\left[2\frac{1}{2}{\left(\pi r_{th}^{2}\right)}^{1/2}\right]=\pi^{5}r_{th}^{10}. \end{array}$$

The result is therefore $N=\pi^{5}r_{th}^{10}$, from which the representation (45) follows and which warrants the normalization condition (51).

The second quantity to consider in view of the calculations of the next sections is the definition of the “temperature” *T*, namely the mean of the variance. This is associated with the positive parameter $r_{th}^{2}$ of the PDF $\rho_{G}\left(g_{L}\left(s_{o}\right),s_{o}\right)$ and is given by a weighted integral in terms of the function ${\left(g\right)}^{2}$ (defined by Equation (46)) as follows:

(55) $$\begin{array}{ccc} T & = & \int_{U_{g}}d\left(g\right){\left(g\right)}^{2}\rho_{G}\left(g_{L}\left(s_{o}\right),s_{o}\right)\\ & = & \frac{1}{N}\underset{ij}{\Pi }\left[2\int_{0}^{+\infty }dg_{\left(i\right)\left(j\right)}{\left(g\right)}^{2}\exp\left\{-\frac{{\left(g\right)}^{2}}{r_{th}^{2}}\right\}\right]\\ & = & \frac{1}{N}\underset{ij}{\Pi }\left[2\frac{1}{2}{\left(\pi r_{th}^{2}\right)}^{1/2}\frac{r_{th}^{2}}{2}\right]=\frac{r_{th}^{20}}{2^{10}}. \end{array}$$

The result shows that the “temperature” *T* is related to the width $r_{th}^{2}$, which represents a familiar conclusion if compared to analogous concepts of statistical physics.

## 6. Determination of the Invariant Parameter $r_{\mathrm{th}}^{2}$: The L(M)-Solution

The quantum-gravity Gaussian PDF introduced above contains the free parameter $r_{th}^{2}$ which measures the width of the Gaussian function and is related to the “temperature” of the distribution. The prescription of the thermal parameter $r_{th}^{2}$ is crucial, as this permits giving physical content to the mathematical solution for the PDF and, in turn, also to the associated entropy functional. In particular, the procedure should relate the expression of $r_{th}^{2}$ to the black-hole invariant properties, in such a way to characterize the quantum PDF to the physical system of interest.

For instance, the same kind of indeterminacy arises also in statistical kinetic and thermodynamical approaches to black-hole entropy [11], where similarly the temperature of the kinetic distribution function for the corresponding statistical distribution remains a free parameter to be prescribed. In these approaches the link to black-holes is met by imposing that the statistical energy content of the kinetic PDF coincides with the mass-energy content of the black-hole, to be assumed composed of the same ensemble of particles with quantum statistical distribution [11]. The method followed in these studies requires the specification of the metric tensor line element, since the particle energy is not formulated as an invariant quantity but is frame-dependent.

It is important to stress, however, that, in principle, the approach to resolve the indeterminacy is not unique. Several alternative possibilities might be proposed, depending on the physical scenario or physical properties to be treated. In this paper, in order to prescribe the thermal width $r_{th}^{2}$ we must bear in mind that, in contrast with phase-space kinetic statistics, the parameter $r_{th}^{2}$ is an invariant 4−scalar and is not related to a thermodynamical temperature, but it measures the variance of the quantum-gravity PDF in the configuration space $U_{g}$. The target is reached by imposing that the quantum expectation value of the nonlinear vacuum Bohm potential $V_{QM}\left(s\right)$ defined by Equation (24), which represents a physical observable, is proportional to the invariant energy $E_{inv}$ associated with the black-hole. Namely, we set the proportionality condition holding between invariant quantities

(56) $$\langle V_{QM}\left(s\right)\rangle \propto E_{inv},$$

to be specified in the calculations below.

The choice of identification of proportionality (56) is motivated by the following considerations:

(1) As explained in Section 3, the definition of the quantum expectation value of arbitrary observables in terms of configuration-space weighted integrals over the PDF $\rho (g_{L}\left(s\right),s)$ according to Equation (38) holds for ordinary continuous tensor functions. In the case of the quantum Hamiltonian density arising in the derivation of the quantum hydrodynamic equations and prescribed by Equation (23), the potential term composed by the effective potential density *V* and the Bohm effective quantum potential $V_{QM}$ satisfies this criterion.
(2) The Bohm potential represents an intrinsic quantum contribution that follows from the introduction of Madelung variables in the quantum wave equation and is not present in the original classical Hamiltonian density before quantization (see Appendix A and Appendix B). At the quantum level, the Bohm potential represents the content of the quantum vacuum that never vanishes, in contrast with the effective potential density *V* that originates from the classical Hamiltonian potential. Therefore, even when V=0 (classical vacuum), one has that $V_{QM}\neq 0$ (quantum vacuum). The quantum nature and the non-vanishing property of $V_{QM}$ support its choice in favor of *V* for the current evaluation of black-hole entropic features.
(3) From the physical point of view, the choice of $V_{QM}$ supports the requirement that both the PDF and the entropic solutions must exist also in the limiting case of vacuum configurations, namely domains external to the black-hole (e.g., a distant observer) where the classical potential is null and only the Hamiltonian quantum vacuum potential remains non-vanishing. It is therefore required that also in this scenario the quantum-gravity PDF preserves the connection with the black-hole state to which the quantum-gravity states belong. The procedure, albeit not unique, provides a concrete example of the complexity of the problem, but also the strength of the theory to gain an analytical solution that effectively permits to characterize the quantum-gravity PDF and the Boltzmann-Shannon entropy to the invariant properties of the black-hole, like its mass, energy and Schwarzschild radius. As such, the quantum Bohm potential is suitably related to the underlying black-hole state through the relationship (56) yields information about the entropic solution in the whole configuration domain, including, necessarily, for physical consistency, the (quantum) vacuum solution.

Let us start evaluating the quantum average of the Bohm potential $V_{QM}\left(s\right)$. First, from the definition (24) we express it in dimensionless form as

(57) $$V_{QM}\left(s\right)=\frac{\hbar^{2}}{\alpha L}U_{QM}\left(s\right),$$

where α=McL and *L* are dimensional constants characteristic of the problem, to be prescribed below. Instead, the function $U_{QM}\left(s\right)$ is given by

(58) $$U_{QM}\left(s\right)=\frac{1}{8}\frac{\partial \ln\rho \left(s\right)}{\partial g^{\mu \nu }}\frac{\partial \ln\rho \left(s\right)}{\partial g_{\mu \nu }}-\frac{1}{4}\frac{\partial^{2}\rho \left(s\right)}{\rho \left(s\right)\partial g_{\mu \nu }\partial g^{\mu \nu }},$$

to be evaluated for the Gaussian PDF $\rho \left(s\right)=\rho_{G}\left(g_{L}\left(s_{o}\right),s_{o}\right)\equiv \rho_{G}\left(s_{o}\right)$ defined by Equation (45). Since

(59) $$\ln\rho_{G}\left(s_{o}\right)=-\ln N-\frac{{\left(g\right)}^{2}}{r_{th}^{2}},$$

then

(60) $$\frac{\partial \ln\rho_{G}\left(s_{o}\right)}{\partial g^{\mu \nu }}\frac{\partial \ln\rho_{G}\left(s_{o}\right)}{\partial g_{\mu \nu }}=\frac{4}{r_{th}^{4}}{\left(g\right)}^{2},$$

so that the first contribution of the Bohm potential gives

(61) $$\frac{1}{8}\frac{\partial \ln\rho_{G}\left(s_{o}\right)}{\partial g^{\mu \nu }}\frac{\partial \ln\rho_{G}\left(s_{o}\right)}{\partial g_{\mu \nu }}=\frac{1}{2}\frac{1}{r_{th}^{4}}{\left(g\right)}^{2}.$$

Instead, the second part of the same potential reads

(62) $$\begin{array}{ccc} \frac{1}{4}\frac{\partial^{2}\rho_{G}\left(s_{o}\right)}{\rho_{G}\left(s_{o}\right)\partial g_{\mu \nu }\partial g^{\mu \nu }} & = & \frac{1}{4\rho_{G}\left(s_{o}\right)}\frac{\partial }{\partial g_{\mu \nu }}\left[\frac{\partial \rho_{G}\left(s_{o}\right)}{\partial g^{\mu \nu }}\right]\\ & = & \frac{1}{4\rho_{G}\left(s_{o}\right)}\frac{\partial }{\partial g_{\mu \nu }}\left[-\frac{2}{r_{th}^{2}}g_{\mu \nu }\rho_{G}\left(s_{o}\right)\right]\\ & = & \frac{1}{4\rho_{G}\left(s_{o}\right)}\left(-\frac{2}{r_{th}^{2}}+\frac{4}{r_{th}^{4}}{\left(g\right)}^{2}\right)\rho_{G}\left(s_{o}\right)\\ & = & -\frac{1}{2r_{th}^{2}}+\frac{1}{r_{th}^{4}}{\left(g\right)}^{2}. \end{array}$$

Hence, the expectation value of $U_{QM}\left(s\right)$ becomes

(63) $$\begin{array}{ccc} \langle U_{QM}\left(s\right)\rangle & = & \int_{U_{g}}d\left(g\right)\left(\frac{1}{2}\frac{1}{r_{th}^{4}}{\left(g\right)}^{2}+\frac{1}{2r_{th}^{2}}-\frac{1}{r_{th}^{4}}{\left(g\right)}^{2}\right)\rho_{G}\left(s_{o}\right)\\ & = & \int_{U_{g}}d\left(g\right)\left(\frac{1}{2r_{th}^{2}}-\frac{1}{2r_{th}^{4}}{\left(g\right)}^{2}\right)\rho_{G}\left(s_{o}\right). \end{array}$$

Recalling the definition of normalization *N* from (54) and the “temperature” from (55) then gives

(64) $$\begin{array}{ccc} \langle U_{QM}\left(s\right)\rangle & = & \frac{1}{2r_{th}^{2}}-\frac{1}{2r_{th}^{4}}\frac{r_{th}^{20}}{2^{10}}\\ & = & \frac{1}{2r_{th}^{2}}-\frac{r_{th}^{16}}{2^{11}}. \end{array}$$

Now, inserting back the dimensional constants from Equation (57), from a dimensional analysis, we can detail the expression (56) as

(65) $$\frac{\hbar^{2}}{\alpha L}\langle U_{QM}\left(s\right)\rangle =K{\left(8\pi \right)}^{2}\frac{E_{inv}}{c},$$

where *K* is a dimensionless proportionality constant and the numerical factor ${\left(8\pi \right)}^{2}$ is included for later convenience. We assume to have a non-rotating Schwarzschild black-hole of mass *M* and Schwarzschild radius $R_{sc}=\frac{2GM}{c^{2}}$, so that the invariant energy $E_{inv}$ is uniquely related to the mass-energy content of the black-hole as

(66) $$E_{inv}=Mc^{2}.$$

In this section, we assign α and *L* in such a way that they are both related to the black-hole mass *M*, namely so that $L=L\left(M\right)$ [34]. We refer to this choice as the $L\left(M\right)-$solution. It is important to emphasize that the choice $L=L\left(M\right)$ has a physical motivation. This permits, in fact, to construct a solution in which the information about the black-hole energy content enters both directly through its mass *M* as well as indirectly through its mass-dependent characteristic length $L\left(M\right)$ (e.g., the Schwarzschild radius, see below). In turn, this introduces a natural re-scaling of the quantum Bohm potential on the characteristic dimensions associated with $L\left(M\right)$. Hence, consistent with the framework set by the $L\left(M\right)$-solution, by specifying the constants $L=L\left(M\right)$ and α as characteristic of black-hole state as

(67) $$\begin{array}{ccc} L & = & R_{sc}, \end{array}$$

(68) $$\begin{array}{ccc} \alpha & = & McR_{sc}, \end{array}$$

we obtain from (65) that

(69) $$\langle U_{QM}\left(s\right)\rangle =K{\left(8\pi \right)}^{2}\frac{M^{2}c^{2}R_{sc}^{2}}{\hbar^{2}},$$

so that, replacing the explicit expression for $R_{sc}$ and introducing the definition of the Planck mass as $m_{P}=\sqrt{\frac{\hbar c}{G}}$ then gives

(70) $$\langle U_{QM}\left(s\right)\rangle ={\left(16\pi \right)}^{2}K\frac{M^{4}}{m_{P}^{4}}.$$

The previous expression can be equivalently expressed in terms of the ratio between the surface area of the black-hole $A=4\pi R_{sc}^{2}$ and the Planck length $\ell_{P}=\sqrt{\frac{G\hbar }{c^{3}}}$ yielding, after simplification of numerical factors, the expression

(71) $$\langle U_{QM}\left(s\right)\rangle =K{\left(\frac{A}{\ell_{P}^{2}}\right)}^{2}.$$

Finally, invoking the expression obtained in (64) for $\langle U_{QM}\left(s\right)\rangle$ gives

(72) $$\frac{1}{2r_{th}^{2}}-\frac{r_{th}^{16}}{2^{11}}=K{\left(\frac{A}{\ell_{P}^{2}}\right)}^{2},$$

which represents an algebraic equation for the unknown variable $r_{th}^{2}$. Its solution provides a representation for $r_{th}^{2}$ in terms of the square of the ratio $\frac{A}{\ell_{P}^{2}}$ of the general form

(73) $$r_{th}^{2}=r_{th}^{2}\left(K{\left(\frac{A}{\ell_{P}^{2}}\right)}^{2}\right),$$

where the proportionality constant must be chosen to ensure that $r_{th}^{2}>0$.

The result provides a physically-based procedure to obtain a representation for the free parameter $r_{th}^{2}$ of the Gaussian quantum PDF that is established in terms of the invariant properties of the black-hole, namely its total mass *M* and characteristic Schwarzschild radius $R_{sc}$ according to the L(M)−solution setting. This completes the determination of the quantum-gravity PDF that can be used to investigate the associated entropy functional.

## 7. Quantum Boltzmann-Shannon Entropy: Dependence on Black-Hole Surface Area in the L(M)−Solution

In terms of the solution of $r_{th}^{2}$ determined in Section 6 it is now possible to evaluate the representation of the Boltzmann-Shannon entropy and its functional dependence on the black-hole surface area, when the choice of the L(M)−solution applies.

The starting point is provided by the representation of the Boltzmann-Shannon entropy given by Equation (44) holding for the initial (or stationary) quantum PDF. Expressing it in terms of the Gaussian PDF, namely letting $\rho (g_{L}\left(s_{o}\right),s_{o})\to$$\rho_{G}\left(g_{L}\left(s_{o}\right),s_{o}\right)\equiv \rho_{G}\left(s_{o}\right)$ we obtain the functional

(74) $$S_{BS}\left(s_{o}\right)=-\int_{U_{g}}d\left(g\right)\rho_{G}\left(s_{o}\right)\ln\rho_{G}\left(s_{o}\right).$$

Inserting the expression (59) for $\ln\rho_{G}\left(s_{o}\right)$ we can write $S_{BS}\left(s_{o}\right)$ as

(75) $$S_{BS}\left(s_{o}\right)=\int_{U_{g}}d\left(g\right)\rho_{G}\left(s_{o}\right)\left[\ln N+\frac{{\left(g\right)}^{2}}{r_{th}^{2}}\right].$$

Then, recalling further the normalization property (51) established above and the definition of the “temperature” for the Gaussian PDF given by Equation (55), we get

(76) $$\begin{array}{ccc} S_{BS}\left(s_{o}\right) & = & \ln N+\frac{1}{r_{th}^{2}}\int_{U_{g}}d\left(g\right){\left(g\right)}^{2}\rho_{G}\left(s_{o}\right)\\ & = & \ln N+\frac{1}{r_{th}^{2}}\frac{r_{th}^{20}}{2^{10}}=\ln N+\frac{r_{th}^{18}}{2^{10}}. \end{array}$$

Since $N=\pi^{5}r_{th}^{10}$ the latter expression can also be written equivalently as

(77) $$S_{BS}\left(s_{o}\right)=5\ln\pi +\ln r_{th}^{10}+\frac{r_{th}^{18}}{2^{10}}.$$

The quantum-gravity Boltzmann-Shannon entropy obtained in this way is an invariant 4−scalar, which depends on the unknown (i.e., free) parameter $r_{th}^{2}>0$, namely the thermal width of the Gaussian quantum PDF. In particular, we can infer the general functional dependence

(78) $$S_{BS}\left(s_{o}\right)=S_{BS}\left(s_{o}\right)\left(r_{th}^{2}\right).$$

Now, if the representation of $r_{th}^{2}$ is established through the procedure detailed in Section 6, then invoking the result provided by Equation (73) yields

(79) $$S_{BS}\left(s_{o}\right)=S_{BS}\left(s_{o}\right)\left(r_{th}^{2}\left(K{\left(\frac{A}{\ell_{P}^{2}}\right)}^{2}\right)\right)\equiv S_{BS}\left({\left(\frac{A}{\ell_{P}^{2}}\right)}^{2}\right),$$

where the last identity must be understood as a short notation underlying the fact that the dependence on the quadratic ratio ${\left(\frac{A}{\ell_{P}^{2}}\right)}^{2}$ is for the thermal radius $r_{th}^{2}$. The quantum Boltzmann-Shannon entropy $S_{BS}\left(s_{o}\right)$ exhibits a specific connection with the black-hole invariant properties inherited by the choice of $r_{th}^{2}$ constructed out of the Bohm potential. For this reason, $S_{BS}\left(s_{o}\right)$ can be regarded as the quantum-gravity Boltzmann-Shannon entropy of a black-hole. It must be remarked that the result has been obtained here by treating only the invariant properties of the black-hole under a very general formalism for the quantum treatment of gravitational field in the framework of CQG-theory and within the validity of the L(M)−solution method. In particular, the entropic properties determined here result from the treatment of the black-hole as a fully quantum object, avoiding approximation schemes or restriction to corresponding surface states only.

We highlight that Equation (79) represents the main physical result. The dependence on the area *A* is a point in common with the Bekenstein-Hawking thermodynamic entropy solution. However, the Boltzmann-Shannon entropy $S_{BS}\left(s_{o}\right)$ depends functionally on the square of the surface-area of the black-hole through the ratio ${\left(\frac{A}{\ell_{P}^{2}}\right)}^{2}$. In the present case the general functional dependence of $S_{BS}\left(s_{o}\right)$ is more involved than that of $S_{BH}$ and ultimately requires the solution of the algebraic Equation (72) to be written explicitly. The profile (79) might mark a point of difference with the Bekenstein-Hawking horizon entropy. However, it must be recalled that $S_{BS}$ arises as a statistical entropy counting black-hole bulk quantum-gravity states rather than horizon states. This follows: (a) from the general mathematical definition of $S_{BS}$ by Equation (42) in terms of quantum-statistic PDF; (b) from the identification of the configuration space for the quantum-gravity tensors with $U_{g}$, which is distinguished from the space-time domain of physical observables; (c) from the prescription of the thermal parameter $r_{th}^{2}$ based on black-hole invariant properties, without needing specification of coordinate systems, event horizon localization or physical phenomena occurring in its surroundings. In this reference, the quadratic dependence in (79) should not be viewed as a contradiction with the semiclassical entropy area law (1), since $S_{BS}$ and $S_{BH}$ should refer to two different application domains, respectively, given by black-hole bulk and surface states. For this reason, at the same time the quadratic area power-law in Equation (79) can provide an interesting possible viable route to the solution of the logical and mathematical issues discussed in Ref. [48] on the connection among entropy, statistical information content and accessible quantum bulk states of a black-hole. On the same ground, the concepts and formalism underlying the CQG Boltzmann-Shannon entropy $S_{BS}$ are not pertinent to holography theory. Hence, $S_{BS}$ should not be understood as a maximal holographic entropy bound arising under the assumption of validity of holographic principle [25], but rather as a microscopic information-statistic measure associated with black-holes quantum-gravity bulk state distributions.

In addition to this, we further notice that the quadratic power-law of $S_{BS}\left(s_{o}\right)$ on the area *A* with respect to the Bekenstein-Hawking linear solution is not unconventional also if compared against the literature. Rather, in view of the discussion provided in the Introduction, it marks a point of contact of the present theory with the findings of alternative classical and quantum approaches to the problem, like for example the Barrow solution [14]. Similarly, the existence of a logarithmic correction term in the entropy representation provided by Equation (77) is in agreement with the outcome generally obtained within alternative quantum-gravity theories and quantum-gravity approaches to the problem of black-hole entropy [29,30,31,32]. The recovery of the logarithmic term also in the framework of CQG-theory strengthens the common belief that such a contribution has a truly peculiar quantum origin.

As a concluding remark, the formalism proposed here has a general character and can be applied to different black-hole scenarios beyond the stationary and spherically symmetric Schwarzschild solution. This can be established by proper evaluation of Equation (57), and in particular the prescription of the corresponding invariant energy $E_{inv}$, which then reflects on the analytical solution for $S_{BS}\left(s_{o}\right)$. In fact, the validity of the approach requires to preserve the invariant character of the physical parameters, which cannot be frame-dependent. In particular, the extension to other black-hole solutions, like the Kerr and Kerr-Newman ones, can be obtained by evaluating $E_{inv}$ for the irreducible black-hole mass by including the appropriate contributions due to rotational and electromagnetic energy.

### Analytical Solution: Lower-Dimensional Case

It is instructive to consider an application of the general formalism developed in the previous sections to treat a physical configuration admitting an analytical solution for both the quantum-gravity PDF and the Boltzmann-Shannon entropy. This is obtained by invoking symmetry conditions on the solutions for both the classical and quantum gravitational tensors and by requiring that the quantum-gravity corrections preserve the same symmetry. As a result, in such a setting it is possible to reduce the dimensions of the quantum degrees of freedom and to treat effectively the problem as if it corresponded to a lower-dimensional case. The conceptual idea at the basis of the procedure is that of taking advantage of spherical symmetry to integrate angular coordinates and yielding in this way an effective theory in 1+1 dimensions corresponding to temporal and radial degrees of freedom. This kind of approach is inspired by an analogy with a procedure implemented in literature studies dedicated to the quantum evaporation phenomena and backreaction of spherically symmetric black-holes. The technique is known as spherical symmetry reduction of dilaton gravity, where generally one introduces an additional field (called dilation) in order to obtain a 2-dimensional dynamical black-hole from the 4-dimensional Einstein theory [72,73,74,75,76,77]. Notice however that the present framework does not require invoking additional auxiliary fields, like the dilaton one, since CQG-theory is self-contained within classical and quantum Hamiltonian theories in terms of the conjugate set of canonical variables $x_{R}\left(s\right)\equiv \left(g\left(s\right),\pi \left(s\right)\right)$. Accordingly, the relationship between the quantum tensor $g_{\mu \nu }$ and the background metric tensor ${\hat{g}}_{\mu \nu }$ is established in terms of background independence and the property of emergent gravity, as proved in Refs. [53,54].

In the current example, let us consider a Schwarzschild space-time metric tensor expressed in spherical coordinates $\left(t,r,\theta ,\phi \right)$ as

(80) $${\hat{g}}_{\mu \nu }=diag\left(-\left(1-\frac{R_{sc}}{r}\right),{\left(1-\frac{R_{sc}}{r}\right)}^{-1},r^{2},r^{2}\sin^{2}\theta \right),$$

and let us require the quantum tensor to have a similar diagonal representation of the type

(81) $$g_{\mu \nu }=diag\left(-A,B,r^{2},r^{2}\sin^{2}\theta \right),$$

where the functions *A* and *B* must be determined consistently in such a way to satisfy the emergent-gravity criterion. Given validity of this ansatz, comparison of Equations (80) and (81) shows that only the radial and temporal components are subject to quantum dynamical effects, while the angular spherical directions of the geometry remain classical. As such, the physical black-hole solution remains correctly defined in the 4−dimensional space-time, while the full quantum-gravitational tensor is similarly expressed by a symmetric rank-4 tensor. Nevertheless, thanks to symmetry conditions we can restrict the general 4−dimensional entropic problem to the 2−dimensional sector of the metric corresponding to dynamical *t* and *r* coordinates. Accordingly, in the following, we refer to this solution as the quantum lower-dimensional case. In order to avoid confusion of notation, we denote the lower-dimensional quantum gravitational tensor with $g_{\alpha \beta }$, where α,β=0,1, preserving the symbol $g_{\mu \nu }$ for the general symmetric tensor with ten independent components.

Let us therefore express the relevant quantities and equations solving for this lower-dimensional case, adopting a separate notation. First of all, we write now the quantum-gravity Gaussian PDF as

(82) $$\rho_{G}\left(g_{L}\left(s_{o}\right),s_{o}\right)=\frac{1}{n}\exp\left\{-\frac{{\left(g\right)}^{2}}{\sigma }\right\},$$

so that the normalization and the “temperature” become respectively

(83) $$\begin{array}{ccc} n & = & {\left(\pi \sigma \right)}^{3/2}, \end{array}$$

(84) $$\begin{array}{ccc} T & = & \frac{\sigma^{3}}{8}. \end{array}$$

The expectation value of $U_{QM}\left(s\right)$ is expressed correspondingly as

(85) $$\begin{array}{ccc} \langle U_{QM}\left(s\right)\rangle & = & \frac{1}{2\sigma }-\frac{1}{2\sigma^{2}}\frac{\sigma^{3}}{8}\\ & = & \frac{1}{2\sigma }-\frac{\sigma }{16}, \end{array}$$

so that the algebraic equation solving for σ is

(86) $$\frac{1}{2\sigma }-\frac{\sigma }{16}=K{\left(\frac{A}{\ell_{P}^{2}}\right)}^{2}.$$

This equation can be solved analytically. To this aim, we preliminary introduce the notation $\gamma \equiv K{\left(\frac{A}{\ell_{P}^{2}}\right)}^{2}$, so that Equation (86) is written as the quadratic equation

(87) $$\sigma^{2}+16\gamma \sigma -8=0,$$

with roots

(88) $$\sigma_{\pm }=-8\gamma \pm \sqrt{64\gamma^{2}+8}.$$

The physically acceptable solution is the positive one $\sigma_{+}>0$, which is found to be

(89) $$\sigma_{+}=-8\gamma +\sqrt{64\gamma^{2}+8}.$$

This carries the predicted functional dependence on the square black-hole surface area $A^{2}$, namely

(90) $$\sigma_{+}=\sigma_{+}\left(\gamma \right).$$

The result proves the correctness of the general derivation developed above and summarized by the validity of Equation (73).

Finally, the Boltzmann-Shannon entropy for the lower-dimensional case treated here gives

(91) $$\begin{array}{ccc} S_{BS}\left(s_{o}\right) & = & \int_{U_{g}}d\left(g\right)\rho_{G}\left(s_{o}\right)\left[\ln n+\frac{{\left(g\right)}^{2}}{\sigma }\right]\\ & = & \ln n+\frac{1}{\sigma }\int_{U_{g}}d\left(g\right){\left(g\right)}^{2}\rho_{G}\left(s_{o}\right)\\ & = & \ln n+\frac{1}{\sigma }\frac{\sigma^{3}}{8}=\ln n+\frac{\sigma^{2}}{8}. \end{array}$$

Recalling the definition of *n* then yields explicitly

(92) $$S_{BS}\left(s_{o}\right)=\frac{3}{2}\ln\pi +\frac{3}{2}\ln\sigma +\frac{\sigma^{2}}{8}.$$

In the limiting case γ≫1 we can expand the root of $\sigma_{+}$ to have

(93) $$\sigma_{+}≃-8\gamma +8\gamma {\left(1+\frac{1}{8\gamma^{2}}\right)}^{1/2}≃\frac{1}{2\gamma },$$

so that the entropy scales asymptotically as

(94) $$\begin{array}{ccc} S_{BS}\left(s_{o}\right) & ≃ & \frac{3}{2}\ln\pi +\frac{3}{2}\ln\frac{1}{2\gamma }+\frac{1}{8}{\left(\frac{1}{2\gamma }\right)}^{2}\\ & = & \frac{3}{2}\ln\frac{\pi }{2}-\frac{3}{2}\ln\gamma +\frac{1}{32}\frac{1}{\gamma^{2}}\\ & ≃ & \frac{3}{2}\ln\frac{\pi }{2}-\frac{3}{2}\ln\gamma . \end{array}$$

It is interesting to interpret this result in the framework of information-statistic point of view and the notion of semiclassical limit. The following considerations apply:

(1) The asymptotic solution (93) can be realized either by setting the limit $\lim_{A\to \infty }\gamma$ acting on the black-hole area, or through the semiclassical limit $\lim_{\hbar \to 0}\gamma$ acting on the Planck length. This gives consistently for the thermal width σ the behavior
  (95) $$\lim_{\hbar \to 0}\sigma =0,$$
  i.e., implying the quantum-gravity PDF to tend to a Dirac-delta, namely
  (96) $$\lim_{\hbar \to 0}\rho_{G}\left(g_{L}\left(s_{o}\right),s_{o}\right)=\delta \left(g_{\alpha \beta }\right).$$
  This means that, correctly, the quantum-gravity Gaussian PDF collapses to the certain classical function in the same classical limit, excluding any quantum statistics in the classical regime.
(2) In the semiclassical limit, we have correspondingly that
  (97) $$\lim_{\hbar \to 0}\gamma =+\infty \Rightarrow \lim_{\hbar \to 0}S_{BS}\left(s_{o}\right)=-\infty ,$$
  so that the Boltzmann-Shannon entropy decreases asymptotically. From the point of view of information statistics, the semiclassical limit corresponds to the quantum probability (or quantum uncertainty) collapsing to the deterministic solution, namely reducing the ignorance on the system or equivalently bringing (classical) order out of (quantum) disorder.
(3) Conversely, the Boltzmann-Shannon quantum entropy increases, evolving from a classical black-hole to a fully quantum black-hole state reached by decreasing the surface area (e.g., through Hawking evaporation phenomena). In fact, when $\gamma ∼O\left(1\right)$, namely for $A∼l_{P}^{2}$, the Boltzmann-Shannon entropy tends to the constant positive value
  (98) $$S_{BS}\left(s_{o}\right)∼\frac{3}{2}\ln\frac{\pi }{2}.$$
  Hence, the quantum-gravity Boltzmann-Shannon entropy reveals itself to be maximum when the black-hole is found in the most quantum state realized at Planck length, and then it decreases when the state tends to a classical regime.

In view of these considerations, we can interpret the quantum-gravity Boltzmann-Shannon entropy in the framework of information statistics as a measure of how close or far the black-hole state is from being regarded and treated as a quantum object rather than a classical one. This follows from the value of the thermal width of the quantum-gravity Gaussian PDF and the knowledge, namely the information, available on its distribution. The determination of this state is based on the expectation value of the quantum-gravity Bohm potential interaction among massive gravitons that permeate the vacuum space-time, preserving the connection with the invariant properties of the black-hole also in domains external to it. The behavior obtained in this way for the Boltzmann-Shannon entropy is the opposite of thermodynamic entropy, because it is related here to the quantum uncertainty of the black-hole to be intended as a quantum-gravity state expressed by the corresponding invariant quantum PDF.

## 8. The Thermodynamic Black-Hole Entropy

In this section, we propose an alternative solution method to establish the expressions for the initial quantum-gravity PDF and the corresponding Boltzmann-Shannon entropy. The discussion is instructive to understand in greater detail on the relationship between quantum-gravity Boltzmann-Shannon and thermodynamic black-hole entropies.

To proceed, we adopt again the Gaussian PDF and the Boltzmann-Shannon entropy both expressed in terms of the free invariant parameter $r_{th}^{2}$. Instead of determining the PDF thermal width using the quantum expectation value of the Bohm potential, and then using the result to express the Boltzmann-Shannon entropy, we follow here a different route. In fact, another possibility could be that of imposing first the condition that the statistical quantum-information Boltzmann-Shannon entropy coincides identically with the thermodynamic black-hole entropy expressed in terms of the black-hole surface law. From this requirement it is possible to resolve the invariant parameter $r_{th}^{2}$ which can then be inserted back into the initial or stationary quantum-gravity Gaussian PDF. As shown below, this alternative procedure generates a physically different solution from that determined in Section 6 and Section 7 above. We must notice in fact that a priori there is no reason for the thermodynamic entropy of space-time to coincide with the Boltzmann-Shannon entropy holding in the g−space. A question to be ascertained in fact is if the thermodynamic entropy for the black-hole coincides or can be properly related to the quantum-gravity statistical-information Boltzmann-Shannon entropy.

Let us start considering the Boltzmann-Shannon entropy in the 10th-dimensional configuration space $U_{g}$ expressed by Equation (77) above, which depends on the free parameter $r_{th}^{2}$. We then impose that the Boltzmann-Shannon entropy coincides with the thermodynamic entropy obtained by Bekenstein-Hawking for a Schwarzschild black-hole given by Equation (1), namely the identity condition

(99) $$S_{BS}\left(s_{o}\right)=S_{BH},$$

We therefore obtain the algebraic equation

(100) $$5\ln\pi +\ln r_{th}^{10}+\frac{r_{th}^{18}}{2^{10}}=\frac{A}{4\ell_{P}^{2}},$$

which can be solved for the invariant parameter $r_{th}^{2}$. The previous equation does not have an analytical solution. However, we can predict the functional dependence

(101) $$r_{th}^{2}=r_{th}^{2}\left(\frac{A}{\ell_{P}^{2}}\right),$$

which shows that this case is different from the solution related to the Bohm potential determined above, see Equation (73).

Again, we can look for an analytical solution to the problem in the lower-dimensional case of 2nd-rank tensor treated above. In this configuration, Equation (100) reduces to

(102) $$3\ln\pi +3\ln\sigma +\frac{\sigma^{2}}{4}=\frac{A}{2\ell_{P}^{2}},$$

which is equivalent to

(103) $$\ln\sigma^{2}+\frac{\sigma^{2}}{6}=\frac{A}{3\ell_{P}^{2}}-2\ln\pi .$$

To obtain an analytical solution, we take the exponential of both sides of the equation yielding

(104) $$\sigma^{2}e^{\frac{\sigma^{2}}{6}}=\exp\left[\frac{A}{3\ell_{P}^{2}}-2\ln\pi \right].$$

Now we introduce the change of variable setting $y=\frac{\sigma^{2}}{6}$, so that Equation (104) reduces to

(105) $$ye^{y}=\frac{1}{6}\exp\left[\frac{A}{3\ell_{P}^{2}}-2\ln\pi \right].$$

This equation is of the type

(106) $$ye^{y}=x,$$

where

(107) $$x\equiv \frac{1}{6}\exp\left[\frac{A}{3\ell_{P}^{2}}-2\ln\pi \right].$$

The solution to Equation (106) can be written by making use of the so-called Lambert *W* function, giving

(108) $$y=W\left[x\right],$$

so that the thermal width is found to be

(109) $$\sigma ={\left(6W\left[x\right]\right)}^{\frac{1}{2}}.$$

We notice that, in this case, the semi-classical limit in which ℏ→0, and therefore also $\ell_{P}^{2}\to 0$, gives the following behavior for σ:

(110) $$\lim_{\hbar \to 0}\sigma =\lim_{\hbar \to 0}{\left(6W\left[\frac{1}{6}e^{\frac{A}{3\ell_{P}^{2}}-2\ln\pi }\right]\right)}^{\frac{1}{2}}=\infty ,$$

which is exactly the opposite of the limit solution (95). From the physical point of view this means that the semiclassical limit in this case does not recover the correct deterministic limit for the quantum PDF. In conclusion, assuming a Gaussian PDF as information-statistic quantum-gravity PDF, it is possible to require and impose the identity between the Boltzmann-Shannon and thermodynamic entropies, determining a solution for the width of the same Gaussian PDF. However, the semiclassical limit of the solution does not recover the deterministic limit of the Gaussian into Dirac-delta function. This prevents the possibility of identifying the thermodynamic black-hole entropy with the Boltzmann-Shannon entropy determined within the framework of manifestly covariant quantum gravity. For this reason, the two approaches remain distinguished and independent, referring to separate physical properties of the system. Nevertheless, the setting proposed here and based on CQG-theory appears as a novel, insightful approach, in principle applicable to other different physical scenarios than the one proposed above.

## 9. Quantum Boltzmann-Shannon Entropy: The $\ell_{P}-$Solution

As a final application of the formalism developed here, we investigate if and how it is possible to reconcile the outcome of Section 6 and Section 7 obtained within the L(M)−solution setting with the proposal of Section 8 on the connection between quantum Boltzmann-Shannon and thermodynamic entropies. From the conclusions of Section 8 it is clear that the exact equal identity between $S_{BS}$ and $S_{BH}$ remains excluded on physical grounds. However, we can nevertheless determine under which conditions the framework provided by CQG-theory could permit obtaining a prediction on the functional dependence of the quantum Boltzmann-Shannon entropy, which is linear in the ratio $\frac{A}{\ell_{P}^{2}}$, rather than quadratic as for the L(M)−solution. We prove below that the result is admissible provided the identification $L\equiv \ell_{P}$ in terms of Planck length is made for the free scale-length of the quantum vacuum Bohm potential. We refer correspondingly to this setting as the $\ell_{P}-$solution, in order to distinguish it from the L(M)−solution.

To proceed with, we invoke again the validity of Equation (65) and the physical motivations at its basis. Then, by specifying the constants *L* and α respectively as

(111) $$\begin{array}{ccc} L & = & \ell_{P}, \end{array}$$

(112) $$\begin{array}{ccc} \alpha & = & Mc\ell_{P}, \end{array}$$

it is immediate to find that $\langle U_{QM}\left(s\right)\rangle$ takes now the form

(113) $$\langle U_{QM}\left(s\right)\rangle =K{\left(8\pi \right)}^{2}\frac{M^{2}G}{\hbar c},$$

or equivalently in terms of the Planck mass $m_{P}$

(114) $$\langle U_{QM}\left(s\right)\rangle =K{\left(8\pi \right)}^{2}\frac{M^{2}}{m_{P}^{2}}.$$

Then, introducing again the notation $A=4\pi R_{sc}^{2}$ for the surface area of the black-hole, after numerical simplification, we arrive at the expression

(115) $$\langle U_{QM}\left(s\right)\rangle =K\left(4\pi \right)\frac{A}{\ell_{P}^{2}}.$$

It follows that in this case the algebraic Equation (72) for the variable $r_{th}^{2}$ depends on the ratio $\frac{A}{\ell_{P}^{2}}$, implying now the formal functional dependence

(116) $$r_{th}^{2}=r_{th}^{2}\left(K\frac{A}{\ell_{P}^{2}}\right),$$

where again the proportionality constant must be chosen to ensure that $r_{th}^{2}>0$.

This result can then be implemented in the representation for the quantum-gravity Boltzmann-Shannon entropy. The validity of Equation (78) implies in this case the general functional dependence

(117) $$S_{BS}\left(s_{o}\right)=S_{BS}\left(s_{o}\right)\left(r_{th}^{2}\left(K\frac{A}{\ell_{P}^{2}}\right)\right)\equiv S_{BS}\left(\frac{A}{\ell_{P}^{2}}\right),$$

where similarly to Section 7, the last identity must be understood as a short notation underlying the fact that the dependence on the linear ratio $\frac{A}{\ell_{P}^{2}}$ is proper of the thermal radius $r_{th}^{2}$.

This calculation proves that also in the framework of CQG-theory, a functional dependence of the quantum-gravity Boltzmann-Shannon entropy on the ratio $\frac{A}{\ell_{P}^{2}}$ can be obtained in analogy with the area-law solution of the Bekenstein-Hawking derivation. The fundamental step that warrants the validity of the result is the identification of the free characteristic scale-length *L* of the vacuum Bohm potential with the invariant universal scale represented by the Planck length $\ell_{P}$. With respect to the L(M)−solution of Section 6 and Section 7, the Planck length is independent of the physical properties of the system of interest, so that the only information about the black-hole contribution to the entropy solution is through its mass *M* and not its characteristic Schwarzschild radius. The choice $L=\ell_{P}$ in fact excludes the possibility of building a model-dependent solution where the black-hole influences both the mass content as well as the length scales of the problem. Nevertheless, the derivation of both the $\ell_{P}-$solution obtained here as well as the $L\left(M\right)-$solution is instructive as it shows the potentiality of the present formalism to explore different physical regimes associated with corresponding black-hole entropy solutions.

## 10. Conclusions

The study of the quantum properties of black-holes represents a crucial topic of theoretical physics and field theory, which poses challenging questions on the fundamentals of our knowledge of General Relativity and Quantum Mechanics. More specifically, this category includes the investigation of entropic aspects and entropic behavior of black-holes as well as their thermodynamic character. The issue is certainly relevant, as it permits to relate microscopic details of the quantum structure of black-holes with their macroscopic features. This concerns therefore the macroscopic manifestation of quantum microscopic dynamical degrees of freedom through invariant black-hole properties that include, in particular, the mass and the peculiar phenomena occurring at the scale-lengths associated with event horizons.

Historically, the basis for the characterization of entropic properties of black-holes dates back to the pioneering works by Hawking and Bekenstein, who discovered the thermodynamical behavior of black-holes and proposed the famous surface-area law for the expression of thermodynamical black-hole entropy. The validity of the surface-area law was then confirmed by means of statistical kinetic theories developed appropriately in the framework of black-hole scenarios with additional validity of suitable assumptions. More recently, the increased interest in the matter has contributed to the proposal of a plethora of alternative and more sophisticated treatments aimed at improving the basic surface-area law to include additional quantum gravitational effects or through alternative competing quantum-gravity approaches.

In this study, the problem has been cast in the framework of manifestly-covariance quantum-gravity theory (CQG-theory), which represents a novel theoretical approach to the formulation of a plausible quantum gravity theory and its application to black-hole entropic physics. In particular, the study of so-called Boltzmann-Shannon entropy of information-statistic theory has been carried out and applied to the context of Schwarzschild’s black-hole scenario. Distinctive features of CQG-theory include the consistency with the principle of manifest covariance and the establishment of a quantum-gravity theory expressed in canonical Hamiltonian form.

Thanks to the probabilistic interpretation rooted on CQG-theory that is analogous to that of Quantum Mechanics, a natural statistical framework can be envisaged that remains associated with the quantum-gravity wave function defined on the corresponding Hilbert space. It is shown that this permits the introduction of appropriate statistical definitions of physical observables determined as configuration-space quantum expectation values weighted over the 4−scalar quantum-gravity probability density function (PDF). In such a setting, it is then possible to postulate a Gaussian profile for the same PDF, which must be appropriately characterized to describe the physical system of interest. More precisely, in the present context, it has been shown that this involves specifying the free parameters of the quantum-gravity PDF, in particular the thermal width of the Gaussian. The resolution of the indeterminacy on the choice of the quantum PDF is not unique and can be met by envisaging different physical criteria that should appropriately relate the quantum solution to the invariant properties of the black-hole, in particular its invariant mass-energy content and the characteristic scale-lengths (e.g., the Schwarzschild radius). As an example, in this paper, this procedure has been established in terms of the mean value of the quantum-gravity nonlinear Bohm vacuum potential. In difference to literature approaches to the same problem developed by kinetic statistical approaches, the novel approach proposed here represents an independent framework that is carried out fully within a canonical quantum-gravity setting of CQG-theory. The latter preserves manifest covariance and does not rely neither on perturbative schemes, nor on symmetry assumptions or the use of external test fields to extrapolate the entropic properties of black-holes. In this derivation, the characteristic free scale-length *L* which normalizes the vacuum Bohm potential has been assumed dependent on the black-hole mass *M*, namely of the type $L=L\left(M\right)$, and more precisely to be identified with its Schwarzschild radius. The corresponding framework has been referred to as the $L\left(M\right)-$solution.

Given the solution for the quantum-gravity PDF appropriate for the treatment of black-holes, a representation for the corresponding Boltzmann-Shannon entropy expressed by an invariant 4−scalar has been established in the $L\left(M\right)-$solution setting. It is proved that this is endowed with a non-trivial functional dependence on the black-hole surface area in terms of the squared ratio ${\left(\frac{A}{\ell_{P}^{2}}\right)}^{2}$. From one side, this recovers a functional dependence on the black-hole surface area, thus excluding other possible black-hole parameters, like the volume, similarly to the Bekenstein-Hawking law. On the other hand, the mathematical expression of the Boltzmann-Shannon entropy is more involved, as it corresponds to the nonlinear quantum dynamics of a symmetric 4−tensor. For this reason, an analtically-tractable example case has been considered to highlight the salient features of the treatment. This corresponds to a lower-dimensional configuration, for which explicit representations for both the Gaussian thermal width and the quantum Boltzmann-Shannon entropy characteristic for the Schwarzschild black-hole have been obtained. On the same ground, the behavior of the solution in the semi-classical limit has been investigated, permitting a critical interpretation of the approach to Boltzmann-Shannon entropy and its logical scheme from the point of view of information statistics.

Finally, the mathematical relationship relating the Boltzmann-Shannon entropy of CQG-theory to the thermodynamical Bekenstein-Hawking entropy has been addressed. It has been proved that it is possible to construct the solution of the Boltzmann-Shannon entropy in such a way that this reproduces exactly the Bekenstein-Hawking entropy area law. However, this generates a corresponding solution for the Gaussian quantum-gravity PDF with unphysical behavior in the semiclassical limit, suggesting that the same solution should be discarded. On the basis of this result, it is then concluded that effectively, the novel approach based on CQG-theory and quantum-gravity Boltzmann-Shannon entropy and the study of entropic black-hole properties remains distinguished and independent from the thermodynamical Bekenstein-Hawking framework. The two entropy functionals, in fact, are found to describe different, but complementary, entropic properties of black-holes, that do not reduce to each other. Interestingly, however, it has been also proved that the same CQG-theory permits investigating an alternative physical regime to the $L\left(M\right)-$solution, which is reached by imposing that *L* is mass-independent and coincides identically with the Planck length. The new outcome obtained under this condition, which is denoted $\ell_{P}-$solution, formally generating a functional dependence of the black-hole entropy on the linear ratio $\frac{A}{\ell_{P}^{2}}$. However, it maintains a separate independent algebraic representation for its explicit solution with respect to the Bekenstein-Hawking area law.

The theory proposed in this paper shows that the complete understanding of the quantum properties of black-holes and their characterization as entropic objects still represent open problems that can stimulate exciting theoretical investigations in the near-future research, either for thermodynamic, statistical kinetic, or quantum gravity and information-statistic settings. Conceptual implications of the present results on the black-hole Boltzmann-Shannon entropy that can be the subject of next investigations include, for example, the study of observable consequences that follow from CQG entropic theory, the implications for the black-hole Hawking evaporation mechanism or the relationship with holography or issues about the information loss paradox. The background formalism established in this paper, together with the analytical solution method proposed, can represent viable frameworks to enhance future studies on such crucial matters.

## Data Availability

Data are contained within the article.

## Appendix A. Synchronous Lagrangian and Hamiltonian Formulations

In this Appendix we provide a summary of the synchronous variational principle based on the deDonder-Weyl formalism for continuum field theory [78]. The synchronous formalism implements a constraint-free manifestly covariant and superabundant variable approach in which the 4−scalar Lagrangian function depends simultaneously on the set of tensor variables $g_{\mu \nu }$ and ${\hat{g}}_{\mu \nu }$. More precisely, in the variational action integral, $g\equiv \left\{g_{\mu \nu }\right\}$ is the variational tensor field (Lagrangian coordinate), which remains distinguished from the non-variational background metric tensor $\hat{g}\equiv \left\{{\hat{g}}_{\mu \nu }\right\}$. This amounts to adopting a background space-time picture in the context of synchronous variational principle to GR, in which the background space-time $\left(Q^{4},\hat{g}\right)$ is considered defined “a priori” in terms of $\hat{g}$, while leaving unconstrained the Lagrangian coordinates *g*. Accordingly, the tensor *g* is an unconstrained symmetric tensor with ten independent components, and therefore it is not subject to gauge properties that characterize the background space-time metric tensor $\hat{g}$. The inclusion of the metric tensor $\hat{g}$ permits the definition of the covariance properties of the theory and, consequently, the prescription of the tensor transformation laws of arbitrary tensor fields. The tensor $\hat{g}$ is assumed to be determined “a posteriori” by the extremal field equations, to be identified with the Einstein field equations, possibly including quantum-gravity effects. Therefore, the metric tensor $\hat{g}$ satisfies both the orthogonality condition ${\hat{g}}_{\mu \nu }{\hat{g}}^{\mu k}=\delta_{\nu }^{k}$, so that it can be used to raise or lower tensor indices, as well as the so-called metric compatibility condition of vanishing covariant derivative ${\hat{\nabla }}_{\alpha }{\hat{g}}_{\mu \nu }=0$, which permits the definition of the standard Christoffel connections. We stress that hereafter all hatted quantities must be understood as being defined with respect to the background metric tensor ${\hat{g}}_{\mu \nu }$. On the contrary, both the inequalities $g_{\mu \nu }g^{\mu k}\neq \delta_{\nu }^{k}$ and ${\hat{\nabla }}_{\alpha }g_{\mu \nu }\neq 0$ apply for the unconstrained variational tensor *g*, which can therefore be characterized by non-vanishing “generalized field velocities” in the Lagrangian function. Since the variational tensor $g_{\mu \nu }$ does not raise/lower indices, the generic covariant variational function $g_{\alpha \beta }$ must be transformed into the corresponding contravariant representation $g^{\alpha \beta }$ by means of the metric tensor ${\hat{g}}_{\alpha \beta }$ only, namely $g_{\alpha \beta }={\hat{g}}_{\alpha \mu }{\hat{g}}_{\beta \nu }g^{\mu \nu }$. Similarly, the “synchronous” definition $d\hat{\Omega }=d^{4}r\sqrt{-\left|\hat{g}\right|}$ is adopted for the invariant 4−volume element, where $d^{4}r$ is the canonical measure of $d\hat{\Omega }$ and $\left|\hat{g}\right|$ denotes the determinant of the metric tensor $\hat{g}$, which implies vanishing synchronous variation, namely $\delta d\hat{\Omega }=0$. It must be stressed, however, that the distinction between *g* and $\hat{g}$ applies only at the level of the variational principle, since the identity $g=\hat{g}$ is recovered in the extremal field equations.

Let us now determine a corresponding manifestly covariant Lagrangian representation for CCG-theory. It is assumed that the 4−scalar Lagrangian function has the following functional form

(A1) $$L\left(Z,\hat{\nabla }Z,\hat{Z}\right)\equiv L\left(Z_{\mu \nu },{\hat{\nabla }}_{\alpha }Z_{\mu \nu },{\hat{Z}}_{\mu \nu }\right),$$

which is taken for completeness to depend on the tensorial variational field $Z\equiv Z_{\mu \nu }$, its covariant derivative $\hat{\nabla }Z\equiv {\hat{\nabla }}_{\alpha }Z_{\mu \nu }$ through at most a quadratic term (first order formalism), and a set of external tensor fields $\hat{Z}\equiv {\hat{Z}}_{\mu \nu }$. The set of Lagrangian variables $\left(Z,\hat{\nabla }Z,\hat{Z}\right)\equiv \left(Z_{\mu \nu },{\hat{\nabla }}_{\alpha }Z_{\mu \nu },{\hat{Z}}_{\mu \nu }\right)$ is then identified with the fields $\left(Z,\hat{\nabla }Z,\hat{Z}\right)\equiv \left(g_{\mu \nu },{\hat{\nabla }}_{\alpha }g_{\mu \nu },{\hat{g}}_{\mu \nu }\right)$. To illustrate the issue, we consider without loss of generality the case of vacuum field equations with non-vanishing cosmological constant. The Lagrangian action functional is denoted by $S_{L}\left(Z,\hat{\nabla }Z,\hat{Z}\right)$, where

(A2) $$S_{L}\left(Z,\hat{\nabla }Z,\hat{Z}\right)=\int d\hat{\Omega }L\left(Z,\hat{\nabla }Z,\hat{Z}\right).$$

Here, the 4−scalar synchronous Lagrangian function $L\left(Z,\hat{\nabla }Z,\hat{Z}\right)$ is expressed as

(A3) $$L\left(Z,\hat{\nabla }Z,\hat{Z}\right)\equiv -\kappa \left[\left(g^{\mu \nu }{\hat{R}}_{\mu \nu }-2\Lambda \right)h-\frac{1}{2}{\hat{\nabla }}^{k}g_{\mu \nu }{\hat{\nabla }}_{k}g^{\mu \nu }\right],$$

in which κ is the dimensional constant $\kappa =\frac{c^{3}}{16\pi G}$, while $h\equiv h\left(Z,\hat{Z}\right)$ is the 4−scalar field

(A4) $$h\left(Z,\hat{Z}\right)\equiv \left(2-\frac{1}{4}g^{\alpha \beta }g_{\alpha \beta }\right).$$

The synchronous Lagrangian variational principle, originally developed in Ref. [78], is then expressed by the identity $\delta S_{L}\left(Z,\hat{\nabla }Z,\hat{Z}\right)=0$ to apply for arbitrary variations of the variational fields *Z*. Here, the operator δ denotes the synchronous variational operator, which is defined in terms of the Frechet derivative and acts in such a way that $\delta \hat{Z}\equiv 0$.

The symbolic Euler-Lagrangian equations corresponding to the variational Lagrangian $L\left(Z,\hat{\nabla }Z,\hat{Z}\right)$ take the manifestly covariant form

(A5) $${\hat{\nabla }}_{i}\frac{\partial L\left(Z,\hat{\nabla }Z,\hat{Z}\right)}{\partial \left({\hat{\nabla }}_{i}g^{\mu \nu }\right)}-\frac{\partial L\left(Z,\hat{\nabla }Z,\hat{Z}\right)}{\partial g^{\mu \nu }}=0,$$

namely, explicitly

(A6) $$\begin{array}{ccc} & & \left.-{\hat{\nabla }}_{i}{\hat{\nabla }}^{i}g_{\mu \nu }-\frac{\partial }{\partial g^{\mu \nu }}\left[\left(g^{\alpha \beta }{\hat{R}}_{\alpha \beta }-2\Lambda \right)h\right]\right.\\ & & \left.+\frac{1}{2}{\hat{\nabla }}^{k}g_{\alpha \beta }{\hat{\nabla }}_{k}g^{\alpha \beta }\frac{\partial }{\partial g^{\mu \nu }}h=0,\right. \end{array}$$

which yield, as a particular realization, the Einstein field equations. The latter ones are recovered when the variational field $g_{\mu \nu }$ is identified with the background tensor ${\hat{g}}_{\mu \nu }$ in the external equation, since then by construction $h\left({\hat{g}}^{\mu \nu }\left(r\right)\right)=1$ and ${\hat{\nabla }}_{\alpha }{\hat{g}}^{\mu \nu }\left(r\right)=0$, so that the previous equation reduces to

(A7) $${\hat{R}}_{\mu \nu }-\frac{1}{2}\hat{R}{\hat{g}}_{\mu \nu }+\Lambda {\hat{g}}_{\mu \nu }=0.$$

The manifestly covariant canonical Hamiltonian theory for the Einstein field equations associated with the Lagrangian formulation given above and expressed in terms of the Lagrangian function $L\left(Z,\hat{\nabla }Z,\hat{Z}\right)$ in Equation (A3) can be readily obtained [79]. In detail, according to the deDonder-Weyl formalism, the canonical momenta are identified from Equation (A5) with the tensor $\Pi_{\mu \nu }^{\alpha }$ defined by

(A8) $$\Pi_{\mu \nu }^{\alpha }=\frac{\partial L\left(Z,\hat{\nabla }Z,\hat{Z}\right)}{\partial \left({\hat{\nabla }}_{\alpha }g^{\mu \nu }\right)}=\kappa {\hat{\nabla }}^{\alpha }g_{\mu \nu }.$$

The corresponding canonical state can then be represented as $\left\{x\right\}=\left\{Z\equiv g^{\mu \nu },\Pi_{\mu \nu }^{\alpha }\right\}$, where, in particular, the tensor $\Pi_{\mu \nu }^{\alpha }$ is symmetric in the lower indices, in the sense that $\Pi_{\mu \nu }^{\alpha }=\Pi_{\nu \mu }^{\alpha }$, and is non-vanishing since for the variational Lagrangian coordinate we have that $g_{\mu \nu }\neq {\hat{g}}_{\mu \nu }$. A remarkable feature of Equation (A8) is that it is invertible, thanks to the quadratic dependence of the Lagrangian with respect to the generalized field velocity. As a consequence, the customary relationship between momenta and generalized velocities occurring in relativistic particle dynamics is recovered. The Hamiltonian $H=H\left(x,\hat{x}\right)$ associated with the Lagrangian $L\left(Z,\hat{\nabla }Z,\hat{Z}\right)$ is then expressed by the Legendre transform

(A9) $$L\left(Z,\hat{\nabla }Z,\hat{Z}\right)\equiv \Pi_{\mu \nu }^{\alpha }{\hat{\nabla }}_{\alpha }g^{\mu \nu }-H\left(x,\hat{x}\right).$$

The latter one can also be conveniently represented in terms of canonical variables $L\left(Z,{\hat{\nabla }}_{\mu }Z,\hat{Z}\right)=L\left(x,\hat{x}\right)$, yielding

(A10) $$L\left(x,\hat{x}\right)=-\kappa h\left[g^{\mu \nu }{\hat{R}}_{\mu \nu }-2\Lambda \right]+\frac{1}{2\kappa }\Pi_{\mu \nu }^{\alpha }\Pi_{\alpha }^{\mu \nu },$$

from which the Hamiltonian $H\left(x,\hat{x}\right)$ follows:

(A11) $$H\left(x,\hat{x}\right)=\frac{1}{2}\frac{1}{\kappa }\Pi_{\mu \nu }^{\alpha }\Pi_{\alpha }^{\mu \nu }+\kappa h\left[g^{\mu \nu }{\hat{R}}_{\mu \nu }-2\Lambda \right].$$

We stress that in the deDonder-Weyl framework realized here, the Lagrangian is regular, so that the transformation from the Lagrangian state to the corresponding Hamiltonian one is non-singular, being realized by a smooth bijection.

As a final step of the derivation, one can introduce the Hamiltonian action functional

(A12) $$\begin{array}{ccc} S_{H}\left(x,\hat{x}\right) & = & \int d\hat{\Omega }L\left(x,\hat{x}\right)\\ & = & \int d\hat{\Omega }\left[\Pi_{\mu \nu }^{\alpha }{\hat{\nabla }}_{\alpha }g^{\mu \nu }-H\left(x,\hat{x}\right)\right], \end{array}$$

where the Lagrangian $L\left(x,\hat{x}\right)$ is given by Equation (A10). Then, imposing validity of the synchronous Hamiltonian variational principle $\delta S_{H}\left(x,\hat{x}\right)=0$ to hold for arbitrary independent variations $\delta g^{\mu \nu }\left(r\right)$ and $\delta \Pi_{\mu \nu }^{\alpha }\left(r\right)$ yields the continuum Hamilton equations

(A13) $$\begin{array}{ccc} \frac{\delta S_{H}\left(x,\hat{x}\right)}{\delta g^{\mu \nu }\left(r\right)} & \equiv & 0⟹{\hat{\nabla }}_{\alpha }\Pi_{\mu \nu }^{\alpha }=-\frac{\partial H\left(x,\hat{x}\right)}{\partial g^{\mu \nu }}, \end{array}$$

(A14) $$\begin{array}{ccc} \frac{\delta S_{H}\left(x,\hat{x}\right)}{\delta \Pi_{\mu \nu }^{\alpha }\left(r\right)} & \equiv & 0⟹{\hat{\nabla }}_{\alpha }g^{\mu \nu }=\frac{1}{\kappa }\Pi_{\alpha }^{\mu \nu }, \end{array}$$

which coincide with the Euler-Lagrange Equation (A6) given above, where in particular the second equation recovers (as usual) the definition of the canonical momentum. When the identification $g_{\alpha \beta }={\hat{g}}_{\alpha \beta }$ is made for external curves, the previous Hamilton equations reduce, respectively, to the Einstein Equation (A7) and the condition ${\hat{\Pi }}_{\mu \nu }^{\alpha }=0$.

## Appendix B. Evolution Form of Hamiltonian Theory

In this Appendix we determine a representation of the manifestly covariant Hamilton equations in evolution form, based on the results and formalism reported in Appendix A (see also Ref. [34]). The latter equations should arise in the framework of a reduced continuum Hamiltonian theory for GR, to be represented by the classical Hamiltonian system $\left\{x_{R},H_{R}\right\}$ formed by a 4−tensor canonical state $x_{R}$ and a 4−scalar Hamiltonian function $H_{R}\left(x_{R},{\hat{x}}_{R}\left(r\right),r,s\right)$. Important requisites to be satisfied by the reduced Hamiltonian theory include: (a) the realization of the reduced variational canonical momentum in terms of a second-rank 4−tensor conjugate to the Lagrangian field variable; (b) the validity of the Einstein field equations, to follow from the corresponding reduced continuum Hamilton equations set in evolution form, to be denoted as GR-Hamilton equations of CCG-theory. More precisely, these must be realized by the initial-value problem represented by the canonical equations:

(A15) $$\left\{\begin{array}{c} \frac{Dg_{\mu \nu }\left(s\right)}{Ds}=\frac{\partial H_{R}\left(x_{R},{\hat{x}}_{R}\left(r\right),r,s\right)}{\partial \pi^{\mu \nu }\left(s\right)},\\ \frac{D\pi_{\mu \nu }\left(s\right)}{Ds}=-\frac{\partial H_{R}\left(x_{R},{\hat{x}}_{R}\left(r\right),r,s\right)}{\partial g^{\mu \nu }\left(s\right)}, \end{array}\right.$$

to be supplemented by initial conditions

(A16) $$\left\{\begin{array}{c} g_{\mu \nu }\left(s_{o}\right)\equiv g_{\mu \nu }^{\left(o\right)}\left(s_{o}\right),\\ \pi_{\mu \nu }\left(s_{o}\right)\equiv \pi_{\mu \nu }^{\left(o\right)}\left(s_{o}\right). \end{array}\right.$$

Here, *s* is the proper-time along an arbitrary geodesic curve $r\left(s\right)\equiv \left\{r^{\mu }\left(s\right)\right\}$ of the background space-time, while

(A17) $$x_{R}\left(s\right)\equiv \left\{g_{\mu \nu }\left(r\left(s\right)\right),\pi_{\mu \nu }\left(r\left(s\right)\right)\right\}$$

is the s−parametrized reduced-dimensional variational canonical state, with $g_{\mu \nu }\left(r\right)$ and $\pi_{\mu \nu }\left(r\right)$ being the corresponding continuous Lagrangian coordinates and the conjugate momenta. It follows that the background state is found to be ${\hat{x}}_{R}\left(s\right)\equiv \left\{{\hat{g}}_{\mu \nu }\left(r\left(s\right)\right),{\hat{\pi }}_{\mu \nu }\left(r\left(s\right)\right)\equiv 0\right\}$. In addition, $\frac{D}{Ds}=\frac{\partial }{\partial s}+t^{\alpha }\left(s\right){\hat{\nabla }}_{\alpha }$ is the covariant s−derivative, with $t^{\alpha }\left(s\right)$ being the tangent 4−vector to the geodesics r(s).

The reduced-dimensional Hamiltonian $H_{R}\left(x,\hat{x},s\right)\equiv H_{R}\left(x_{R},{\hat{x}}_{R}\left(r\right),r,s\right)$ follows from Equation (A11) and is represented as

(A18) $$H_{R}\left(x,\hat{x},s\right)\equiv \frac{1}{2}\frac{1}{\kappa }\pi_{\mu \nu }\pi^{\mu \nu }+\kappa h\left[g^{\mu \nu }{\hat{R}}_{\mu \nu }-2\Lambda \right],$$

where the canonical momenta $\Pi_{\mu \nu }^{\alpha }$ are replaced by the reduced-dimensional ones $\pi_{\mu \nu }$ in the “kinetic” term of the Hamiltonian. The connection between the Hamiltonian system $\left\{x,H\right\}$ expressed by the canonical state $x\equiv \left\{g^{\mu \nu },\Pi_{\mu \nu }^{\alpha }\right\}$ and the “reduced” Hamiltonian system $\left\{x_{R},H_{R}\right\}$ is obtained upon identifying $\Pi_{\mu \nu }^{\alpha }=t^{\alpha }\pi_{\mu \nu }$, from which it follows that $\pi_{\mu \nu }\left(r\right)$ arises from the projection of $\Pi_{\mu \nu }^{\alpha }\left(r\right)$ along $t_{\alpha }\left(s\right)$ as

(A19) $$\pi_{\mu \nu }\equiv t_{\alpha }\Pi_{\mu \nu }^{\alpha }.$$

A trajectory-based parametrization of the Hamiltonian state can be given based on the notion of Lagrangian path [80]. The latter is defined by the geodetic curve $\left\{r^{\mu }\left(s\right)\right\}\equiv \left\{\left.r^{\mu }\left(s\right)\right|\;\forall s\in R,\;r^{\mu }\left(s_{o}\right)=r_{o}^{\mu }\right\}$ having $t^{\gamma }\left(\hat{g}\left(r\right),r\right)$ as the tangent 4−vector at an arbitrary 4−position $r\equiv \left\{r^{\mu }\right\}$ of the space-time $\left(Q^{4},\hat{g}\left(r\right)\right)$ [35]. The proper-time parametrization is then obtained by replacing $r\equiv \left\{r^{\mu }\right\}$ with $r\left(s\right)\equiv \left\{r^{\mu }\left(s\right)\right\}$, namely, by setting

(A20) $$\left\{\begin{array}{c} g^{\mu \nu }\left(s\right)\equiv g^{\mu \nu }\left(r\left(s\right)\right),\\ \pi^{\mu \nu }\left(s\right)\equiv \pi^{\mu \nu }\left(r\left(s\right)\right),\\ \hat{x}\left(r\right)\equiv \hat{x}\left(r\left(s\right)\right). \end{array}\right.$$

A reduced Hamiltonian variational principle yielding Equation (A15) and expressed in terms of the reduced Hamiltonian state can be developed. The variational functional in such a case is identified with the real 4−scalar

(A21) $$S_{R}\left(x,\hat{x}\right)\equiv \int d\hat{\Omega }L_{R}\left(x,\hat{x},r,s\right),$$

where the variational Lagrangian $L_{R}\left(x,\hat{x},r,s\right)$ is the Legendre transform of the corresponding variational Hamiltonian $H_{R}\left(x,\hat{x},r,s\right)$:

(A22) $$L_{R}\left(x,\hat{x},r,s\right)\equiv \pi_{\mu \nu }\frac{D}{Ds}g^{\mu \nu }-H_{R}\left(x,\hat{x},r,s\right).$$

The synchronous variational principle for the functional $S_{R}\left(x,\hat{x}\right)$ is written as

(A23) $$\delta S_{R}\left(x,\hat{x}\right)=0,$$

in which both the state $\hat{x}$ and the 4−scalar volume element $d\hat{\Omega }$ must be treated as non-variational by definition. This generates 4−tensor Euler-Lagrange equations which are equivalent to the Hamilton Equation (A15). When cast in symbolic form they read as

(A24) $$\left\{\begin{array}{c} \frac{\delta S_{R}\left(x,\hat{x}\right)}{\delta g^{\mu \nu }}=0,\\ \frac{\delta S_{R}\left(x,\hat{x}\right)}{\delta \pi_{\mu \nu }}=0. \end{array}\right.$$

These equations can be written also in the equivalent Poisson-bracket notation as

(A25) $$\frac{D}{Ds}x_{R}\left(s\right)={\left[x_{R},H_{R}\left(s\right)\right]}_{\left(x_{R}\right)},$$

where the Poisson bracket ${\left[,\right]}_{\left(x_{R}\right)}$ is defined as

(A26) $${\left[x_{R},H_{R}\left(s\right)\right]}_{\left(x_{R}\right)}=\frac{\partial x_{R}}{\partial g^{\mu \nu }}\frac{\partial H_{R}\left(s\right)}{\partial \pi_{\mu \nu }}-\frac{\partial x_{R}}{\partial \pi_{\mu \nu }}\frac{\partial H_{R}\left(s\right)}{\partial g^{\mu \nu }}.$$

We notice that Equation (A25) yield exactly the GR-Hamilton equations in evolution form expressed by Equation (A15). In fact, by implementing the explicit representation of $H_{R}\left(s\right)$ given by (A18) gives

(A27) $$\frac{\partial H_{R}\left(s\right)}{\partial g^{\mu \nu }\left(s\right)}=\kappa h\left(s\right){\hat{R}}_{\mu \nu }-\kappa g_{\mu \nu }\left(s\right)\frac{1}{2}\left(g^{\alpha \beta }\left(s\right){\hat{R}}_{\alpha \beta }\right).$$

The canonical Equation (A15) then reduce to the single equivalent Lagrangian–path parametrized evolution equation for $g_{\mu \nu }\left(s\right)$:

(A28) $$\frac{D}{Ds}\left[\frac{D}{Ds}g_{\mu \nu }\left(s\right)\right]+h\left(s\right){\hat{R}}_{\mu \nu }-g_{\mu \nu }\left(s\right)\frac{1}{2}\left[g^{\alpha \beta }\left(s\right){\hat{R}}_{\alpha \beta }-2\Lambda \right]=0.$$

The final issue to address concerns the proof of the relationship between the canonical Equation (A15) and GR theory. This is obtained assuming that the Hamiltonian does not depend explicitly on proper-time *s* and is therefore of the form $H_{R}=H_{R}\left(x_{R},{\hat{x}}_{R}\left(r\right),r\right)$. Then, since ${\hat{g}}_{\mu \nu }\left(s\right){\hat{g}}^{\mu \nu }\left(s\right)=\delta_{\mu }^{\mu }$ and $\frac{D}{Ds}{\hat{g}}_{\mu \nu }\left(s\right)\equiv 0$, also ${\hat{\pi }}_{\mu \nu }\left(s\right)\equiv 0$, so that the canonical equation for ${\hat{\pi }}_{\mu \nu }\left(s\right)$ (or equivalently Equation (A28)) yields

(A29) $${\hat{R}}_{\mu \nu }-{\hat{g}}_{\mu \nu }\left(s\right)\frac{1}{2}{\hat{g}}^{\alpha \beta }\left(s\right){\hat{R}}_{\alpha \beta }+\Lambda {\hat{g}}_{\mu \nu }\left(s\right)=0,$$

which coincides with the Einstein field Equation (A7). Therefore, the latter arises as a stationary solution of the GR-Hamilton Equation (A15) with respect to proper-time, i.e., imposing the initial conditions

(A30) $$\left\{\begin{array}{c} g_{\mu \nu }\left(s_{o}\right)\equiv {\hat{g}}_{\mu \nu }\left(s_{o}\right),\\ \pi_{\mu \nu }\left(s_{o}\right)\equiv {\hat{\pi }}_{\mu \nu }\left(s_{o}\right)=0, \end{array}\right.$$

together with the identity ${\hat{\pi }}_{\mu \nu }\left(s\right)=0$ for all s∈I.
